# Comparison of the efficacy of 12 interventions in the treatment of diabetic foot ulcers: a network meta-analysis

**DOI:** 10.7717/peerj.19809

**Published:** 2025-08-11

**Authors:** Xuyang Hu, Huixin Meng, Jiaru Liang, Hang An, Jiaqi Zhou, Yuling Gao, Chong You, Zhenni Zhang, Xiaoyang Gong, Yong Liu

**Affiliations:** 1 Department of Rehabilitation Medicine, The First Affiliated Hospital of Dalian Medical University, Dalian, China; 2 Institute (College) of Integrative Medicine, Dalian Medical University, Dalian, China

**Keywords:** Diabetic foot ulcers, Network meta-analysis, Wound healing rate, Wound healing time, Percentage area reduction, Amputation rate

## Abstract

**Objective:**

This study aimed to comprehensively compare the efficacy of 12 interventions for diabetic foot ulcer (DFU) using a network meta-analysis (NMA).

**Methods:**

The NMA was conducted by PRISMA guidelines, and the protocol was registered in PROSPERO (CRD42023461811). PubMed, Web of Science, Cochrane Library, and Embase databases were systematically searched from inception to September 2023. Randomized controlled trials (RCTs) enrolling patients with DFU were included if they compared epidermal growth factor (EGF), platelet-derived growth factor (PDGF), platelet-rich plasma (PRP), stem cells (SC), low-frequency ultrasound (LFU), negative pressure wound therapy (NPWT), low-level laser therapy (LLLT), electric stimulation (ES), extracorporeal shockwave therapy (ESWT), amniotic membrane therapy (AMT), hyperbaric oxygen therapy (HBOT), and topical oxygen therapy (TOT) against standard of care (SOC) or placebo. The primary endpoint assessed was the wound healing rate. Secondary endpoints comprised wound healing time, percentage area reduction (PAR), and amputation rate. The surface under the cumulative ranking curve (SUCRA) was calculated to rank the efficacy of interventions.

**Results:**

A total of 99 RCTs involving 7,356 patients were included. Among the 12 interventions analyzed, only LFU (OR = 2.20; 95% CI [0.99–4.91]) and ES (OR = 1.88; 95% CI [0.87–4.05]) did not demonstrate statistically significant improvements in ulcer healing rate compared with SOC. Based on SUCRA rankings, SC (SUCRA = 89.7%; OR = 5.71; 95% CI [2.64–12.34]) and AMT (SUCRA = 89.2%; OR = 5.11; 95% CI [3.12–8.37]) ranked highest in promoting ulcer healing, while LFU (29.4%) and SOC (10.4%) ranked lowest. Regarding wound healing time, AMT (MD = –26.91 days; 95% CI [−44.27 to −9.55]), PRP (MD = –21.65 days; 95% CI [−33.61 to −9.69]), and NPWT (MD = −16.79 days; 95% CI [−31.12 to −2.26]) significantly reduced healing durations compared to SOC. SUCRA rankings indicated that AMT (84.7%) and PRP (74.6%) ranked highest, while LFU (29.4%) and SOC (10.4%) remained lowest. Concerning PAR, LLLT (MD = 34.27; 95% CI [17.35–51.20]) and ESWT (MD = 27.50; 95% CI [11.00–44.00]) showed significant improvements over SOC, with LLLT (SUCRA = 93.9%) and ESWT (SUCRA = 84.0%) ranking highest, while SOC (21.0%) and TOT (18.3%) ranked lowest. For amputation rate, SC (OR = 0.12; 95% CI [0.03–0.55]) and HBOT (OR = 0.35; 95% CI [0.16–0.78]) significantly lowered the risk compared to SOC, with SUCRA rankings placing SC (79.9%) and PRP (73.2%) as most effective, while NPWT (26.4%) and SOC (9.9%) were least effective.

**Conclusions:**

SC and AMT emerged as highly effective, demonstrating superior efficacy in improving healing rate compared to PDGF, ES, and HBOT. AMT also showed significant effects in shortening ulcer healing time. LLLT exhibited considerable effectiveness in reducing ulcer areas, and SC therapy was associated with reduced amputation rate.

## Introduction

Diabetes has become one of the most serious and common diseases in the world. In 2021, diabetes affected 10.5% of the global population aged 20 to 79 years, corresponding to approximately 536.6 million individuals. This prevalence is anticipated to increase to 12.2%, impacting around 783.2 million people by the year 2045 (Sun et al., 2022a). Diabetic foot ulcer (DFU) is a serious complication of diabetes, characterized by skin breakdown in the feet, involving at least the epidermis and part of the dermis, usually accompanied by lower limb neuropathy and/or peripheral arterial disease (Van Netten et al., 2024). Epidemiological studies suggest that 19–34% of diabetic patients will suffer from DFU, about 60% of DFU patients will develop infection, about 20% will require lower limb amputation, and 10% will die within the first year of diagnosis (McDermott et al., 2023). DFU imposes a substantial economic burden on healthcare systems and individual expenses, amounting to US$9 to 13 billion annually, which is two to three times higher than that for non-DFU patients (Rice et al., 2014). The development of DFU is mainly associated with four aspects: ischemia caused by peripheral arterial disease, leading to tissue necrosis due to insufficient blood supply; peripheral neuropathy leads to sensory, motor and secretory dysfunction in the lower limb skin, resulting in loss of protection and delayed healing; chronic inflammation caused by bacterial infection also delays healing and cell dysfunction hinders wound repair (Deng et al., 2023a). The normal wound healing process includes four phases: hemostasis, inflammation, proliferation, and remodeling (Mamun et al., 2024). However, in diabetic wounds, these programmed phases are disrupted, preventing normal wound healing. This complex pathological process brings unique challenges to the management of DFU, necessitating advanced therapeutic strategies to enhance patient outcomes.

Currently, the clinical management of DFU primarily relies on standard of care (SOC), including local debridement, wound dressing, offloading, revascularization, and infection control (Schaper et al., 2024). These approaches greatly relieve symptoms and promote wound healing, constituting a fundamental clinical strategy. Over the past decades, a variety of advanced interventions have been explored to improve DFU healing. Such interventions encompass a range of therapies: epidermal growth factor (EGF), platelet-derived growth factor (PDGF), platelet-rich plasma (PRP), stem cells (SC), low-frequency ultrasound (LFU), negative pressure wound therapy (NPWT), low-level laser therapy (LLLT), electric stimulation (ES), extracorporeal shockwave therapy (ESWT), amniotic membrane therapy (AMT), hyperbaric oxygen therapy (HBOT), and topical oxygen therapy (TOT) (Armstrong et al., 2023; Ganesan & Orgill, 2024). These therapies have shown promising potential. Several randomized controlled trials (RCTs) have demonstrated promising efficacy for these interventions. For instance, AMT, SC therapy, and LLLT have revealed improved wound healing rate, shortened healing time, and reduced wound area (DiDomenico et al., 2018; Haze et al., 2022; Uzun et al., 2021). Additionally, multiple meta-analyses have confirmed that these treatments are superior in terms of healing rate, area reduction, and amputation prevention. A meta-analysis of nine RCTs involving 720 participants confirmed that EGF significantly improved the healing rate compared to SOC (OR: 2.79; 95% CI [1.99–3.99]) and significantly shortened healing time (MD: −14.10 days; 95% CI [−18.03 to −0.16]) (Zhao et al., 2020). Similarly, a meta-analysis of 22 RCTs (1,559 patients) revealed that PRP significantly enhanced the healing rate (RR: 1.42; 95% CI [1.30–1.56], *P* < 0.001), and reduced healing time (MD: −3.13 days; 95% CI [−5.86 to −0.39], *P* < 0.001) compared with SOC (Deng et al., 2023b). In addition, SC therapy, LLLT, ES, NPWT, AMT, HBOT, and TOT have been associated with significant enhancements in healing rate, along with a decrease in ulcer size and a lower risk of amputation across multiple RCTs (Chen et al., 2021; Chen et al., 2020; Huang et al., 2021; Huang et al., 2023; Mohammed et al., 2022; Sun et al., 2022b; Tao & Yuan, 2024).

Despite promising findings from individual interventions, existing guidelines highlight variations in the effectiveness of different treatments for DFU (Chen et al., 2024). Most published meta-analyses in the field of DFU have adopted traditional pairwise comparison methods. However, with the increasing number of clinical interventions for DFU, pairwise comparisons alone are insufficient to meet the needs of comprehensive evaluations. To date, two network meta-analyses (NMAs) have compared more than three interventions (OuYang et al., 2024; Qian et al., 2024). However, the interventions included were not comprehensive, omitting several commonly used clinical treatments. Therefore, this study aims to employ NMA, which enables indirect comparisons through a common control group, providing a more comprehensive evaluation of relative effectiveness (Salanti et al., 2008), to compare the effectiveness of 12 interventions for DFU.

### Methods

This NMA adhered to the Preferred Reporting Items for Systematic Reviews and Meta-Analyses (PRISMA) guidelines. To ensure greater transparency and reproducibility of the research, the study protocol was prospectively registered in the PROSPERO database (CRD42023461811).

### Data sources and search strategies

Two authors (HXY and MHX) independently searched PubMed, Web of Science, Cochrane Library, and Embase from inception to September 2023, utilizing both Medical Subject Headings (MeSH) and free-text terms. As NMA requires data from at least three RCTs, we identified 13 additional intervention types beyond the SOC through preliminary searches. The complete search strategies for each database, including detailed search terms, are provided in Supplemental Information 2.

### Study selection

We included RCTs evaluating 12 interventions for treating patients diagnosed with DFU, including EGF, PDGF, PRP, SC, LFU, NPWT, LLLT, ES, ESWT, AMT, HBOT, and TOT. Studies were eligible if the control group received SOC or a placebo. Studies comparing two or more interventions were included if they met the other criteria. Included studies were required to report the outcome of wound healing rate, wound healing time, percentage area reduction (PAR), or amputation rate, including both major and minor amputations. Eligible studies needed to clearly define and report these outcome measures and be published in English.

We excluded studies involving patients without DFU, non-RCTs, and studies that lacked clearly defined outcome measures. Studies with fewer than 10 participants or a total study duration shorter than 4 weeks (from intervention initiation to final assessment) were also excluded. Of the 13 interventions identified during the preliminary search, 12 were included in the final analysis because the number of eligible RCTs investigating ozone therapy was inadequate to satisfy the inclusion criteria.

### Data extraction and quality assessment

Two authors, MHX and LJR, independently extracted data from the included RCTs. Any discrepancies were reconciled through consensus with the involvement of a third reviewer, HXY. The extracted data included study characteristics (author, year, country, and sample size) and population demographics (age, gender, ulcer grade, and treatment duration). Additionally, details of interventions, control measures, and clinical outcomes were collected. Two authors, YC and AH, evaluated the risk of bias using the Cochrane tool, focusing on factors such as random sequence generation, allocation concealment, and blinding. In instances of disagreement, consensus was achieved through discussion and subsequently adjudicated by a third author, HXY.

### Data analysis

We conducted a frequentist network meta-analysis using a random-effects model to simultaneously estimate the relative effects of all interventions by combining both direct and indirect evidence (Higgins et al., 2012). To ensure network connectivity and coherent synthesis, trials were aggregated into 12 intervention nodes according to their principal therapeutic modality (*e.g.*, growth-factor, physical, or cellular therapies). Analyses were conducted at the node level, assuming that treatments within each node share similar mechanisms of action and clinical intent, consistent with the transitivity assumption in NMA (Cornell, 2015). We assessed network consistency and overall heterogeneity in all treatment comparisons. Global inconsistency tests and the node-splitting method were used to evaluate disparities between direct and indirect evidence, with a *p*-value < 0.05 indicating significant heterogeneity or inconsistency. All statistical analyses were performed using STATA 16.0. Interventions were ranked based on their effect sizes by calculating Surface Under the Cumulative Ranking (SUCRA) values. We explicitly defined the primary and secondary outcomes to assess the effectiveness of treatments on DFU healing. The primary endpoint assessed was the wound healing rate. Secondary endpoints comprised wound healing time, PAR, and amputation rate. Dichotomous variables were reported as odds ratios (OR) with 95% confidence intervals (CI), whereas continuous variables were expressed as mean differences (MD) with 95% CI. We conducted subgroup analyses to evaluate whether treatment effects varied based on study duration. Separate network meta-analyses were conducted for each outcome, stratified by follow-up duration (≤12 weeks *vs.* >12 weeks).

## Results

### Study characteristics

**Figure 1:**
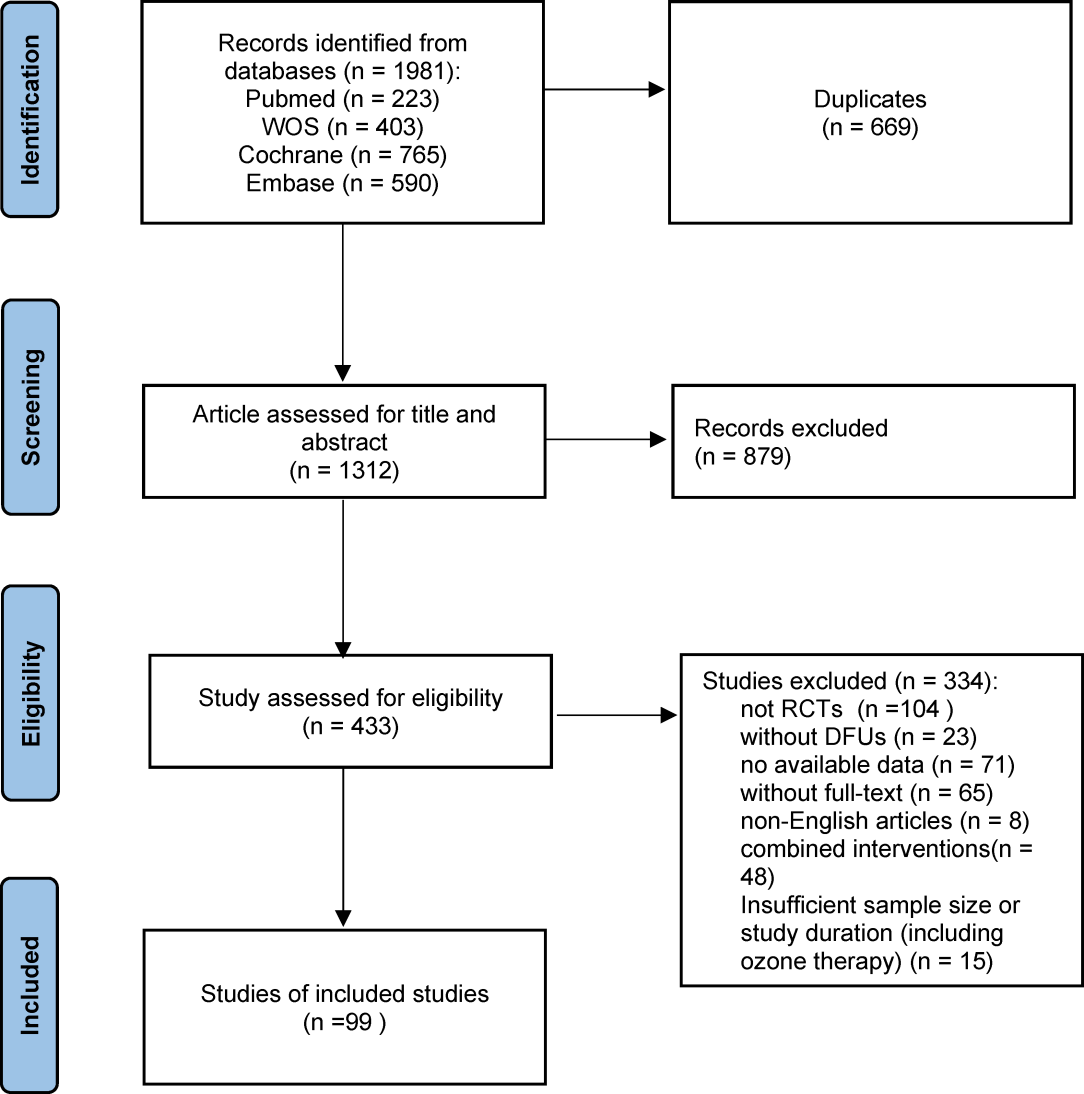
Flow chart of the study selection process. A total of 1,981 records were identified from four databases. After removing duplicates and screening titles, abstracts, and full texts, 99 RCTs were included in the final analysis.

During our systematic search, we identified 1,981 references, of which 669 were duplicates, leaving 1,312 for detailed review. After the initial assessment of titles and abstracts, 879 articles were removed. Subsequently, 433 articles underwent full-text review for eligibility, and 319 studies were excluded based on predefined criteria, including non-DFU populations, lack of randomization, unavailable full-text data, and the use of combined interventions. An additional 15 studies were excluded based on insufficient duration and small sample sizes. Ultimately, 99 studies met the eligibility criteria and were included in the final analysis. The PRISMA flowchart (Fig. 1) illustrates the detailed selection process.

**Figure 2:**
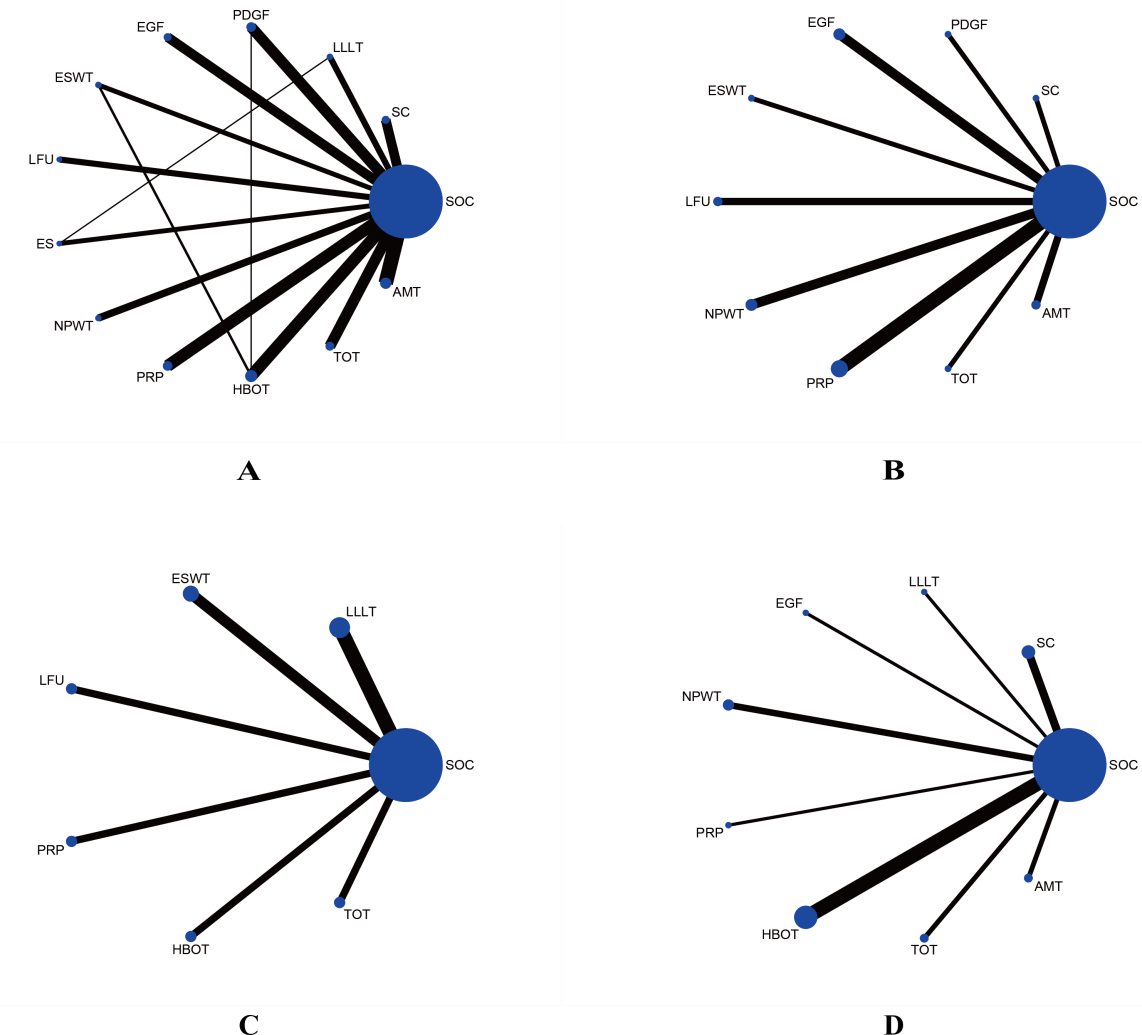
Network plots comparing different interventions for four outcomes. (A) Wound healing rate; (B) wound healing time; (C) percentage area reduction (PAR); (D) amputation rate. The blue nodes represent individual interventions, with node size proportional to the sample size of each intervention. Lines between nodes indicate direct comparisons from included RCTs, with line thickness proportional to the number of studies performing each comparison.

The included RCTs were primarily conducted in the USA, India, and China, with publication years ranging from 1992 to 2023. A total of 7,356 participants were enrolled, with sample sizes ranging from eight to 382 and participant ages spanning from 32 to 72 years. Detailed study characteristics are summarized in Table 1. These RCTs assessed a variety of treatments, including 10 comparing EGF with SOC (Afshari et al., 2005; Fernández-Montequín et al., 2009; Gomez-Villa et al., 2014; Oliveira et al., 2021; Park et al., 2018; Singla et al., 2014; Tsang et al., 2003; Viswanathan, Juttada & Babu, 2020; Xu et al., 2018; Zhang et al., 2021a), nine studies on PDGF (Agrawal et al., 2009; Bhansali et al., 2009; Blume et al., 2011; Jaiswal et al., 2010; Khandelwal et al., 2013; Ma et al., 2015; Samuel et al., 2016; Steed, 1995; Wieman, Smiell & Su, 1998), 11 on PRP (Ahmed et al., 2017; Driver et al., 2006; Elsaid et al., 2020; Gowsick et al., 2023; Gupta et al., 2021; Hossam et al., 2022; Li et al., 2015; Malekpour Alamdari et al., 2021b; Orban et al., 2022; Singh et al., 2018; Xie et al., 2020), eight on SC Therapy (Lu et al., 2008; Han, Kim & Kim, 2010; Huang et al., 2005; Jain et al., 2011; Lu et al., 2011; Mohammadzadeh et al., 2013; Ozturk et al., 2012; Uzun et al., 2021), five on LFU (Abd El Fattah et al., 2018; Ennis et al., 2005; Lázaro-Martínez et al., 2020; Michailidis et al., 2018; Rastogi, Bhansali & Ramachandran, 2019), eight on NPWT (Anjum et al., 2022; Blume et al., 2008; Karatepe et al., 2011; Malekpour Alamdari et al., 2021a; Maranna et al., 2021; Nain et al., 2011; Seidel, Lefering & DiaFu study, 2022; Seidel et al., 2020), six on LLLT (Haze et al., 2022; Kaviani et al., 2011; Minatel et al., 2009; De Alencar Fonseca Santos et al., 2018; Naidu et al., 2005), three on ES Therapy (Baker et al., 1997; Lundeberg, Eriksson & Malm, 1992; Peters et al., 2001), eight on ESWT (Jeppesen et al., 2016; Moretti et al., 2009; Nossair, Eid & Salama, 2013; Omar et al., 2014; Sandoval Ortíz et al., 2014; Snyder et al., 2018; Vangaveti et al., 2023; Wang et al., 2009; Wang, Wu & Yang, 2011), 12 on AMT (DiDomenico et al., 2018; Game et al., 2021; Lavery et al., 2014; Mohajeri-Tehrani et al., 2016; Serena et al., 2020; Snyder et al., 2016; Tettelbach et al., 2019a; Tettelbach et al., 2019b; Thompson et al., 2019; Zelen et al., 2015; Zelen et al., 2013; Zelen et al., 2016), 10 on HBOT (Abidia et al., 2003; Chen et al., 2017; Duzgun et al., 2008; Faglia et al., 1996; Fedorko et al., 2016; Kessler et al., 2003; Kumar et al., 2020; Londahl et al., 2010; Salama et al., 2019; Santema et al., 2018), and nine on TOT (Al-Jalodi et al., 2022; Driver et al., 2017; Frykberg et al., 2020; He et al., 2021; Niederauer, Michalek & Armstrong, 2015; Niederauer, Michalek & Armstrong, 2017; Niederauer et al., 2018; Serena et al., 2021; Yu et al., 2016). Figure 2 illustrates the treatment network for the various interventions.

**Table 1:** Characteristics of the included studies.

**Author year**	**Country**	**Size I/C**	**Gender distribution M/F**	**Comparisons**	**Mean age (years)**		**Study duration**	**Treatment duration**	**Ulcer grade**	**Outcomes**
Han 2010	Korea	26/26	29/23	SC *vs* SOC	66.5	68.4	8 weeks	8 weeks	Wagner: 1–2	①②
Huang 2005	China	14/14	18/10	SC *vs* SOC	71.1	70.9	12 weeks	—	TEXAS: 1–2C, 1–3D	①④
Jain 2011	India	25/23	32/16	SC *vs* SOC	54	58	12 weeks	—	—	①
Lu 2008	China	22/23	26/19	SC *vs* SOC	66.5	65.52	12 weeks	—	Fontaine grade IV	①④
Lu 2011	China	41/41	—	SC *vs* SOC	64	64	24 weeks	—	—	①④
Mohammadzadeh 2013	Iran	7/14	—	SC *vs* SOC	63.5	64.2	12 weeks	—	—	④
Ozturk 2012	Turkey	20/20	16/13	SC *vs* SOC	71.9	70.8	12 weeks	—	—	①
Uzun 2021	Turkey	10/10	12/8	SC *vs* SOC	57.5	57.2	4 years	—	Wagner: 1–2	①②④
Haze 2021	Israel	10/10	13/7	LLLT *vs* SOC	65	61	12 weeks	12 weeks	TEXAS: IIA, IIIA/B,	①③④
Kaviani 2011	Iran	13/10	12/6	LLLT *vs* SOC	60.2	59.4	20 weeks	20 weeks	Wagner: 1–2	①③④
Minatel 2009	USA	12/11	—	LLLT *vs* SOC	66.3	63.4	12 weeks	12 weeks	—	①③
Naidu 2005	Malaysia	8/8	—	LLLT *vs* SOC	40–50		6 weeks	6 weeks	with “Depth- Ischaemia Classification” grade 1 foot ulcers	①
Sandoval Ortíz 2014	Colombia	9/10/9	12/16	LLLT *vs* ES *vs* SOC	59.3		16 weeks	16 weeks	Wagner: 1–2	①
de Alencar Fonseca Santos 2018	Brazil	9/9	—	LLLT *vs* SOC	53.11	48.33	4 weeks	4 weeks	Noninfected lesion with II and III depth	③
Agrawal 2009	India	14/14	19/9	PDGF *vs* SOC	54.38	56.24	12 weeks	12 weeks	Wagner: 1-4	①
Bhansali 2009	India	10/10	12/8	PDGF *vs* SOC	51.7	49.5	20 weeks	20 weeks	Wagner: >2	①②
Blume 2011	USA	72/19	65/26	PDGF *vs* SOC	57.9	54.8	12 weeks	12 weeks	Wagner: 1	①
Jaiswal 2010	India	25/25	42/8	PDGF *vs* SOC	56.20	49.92	10 weeks	10 weeks	IAET stage III and IV	①
Khandelwal 2013	India	20/20/20	32/28	PDGF *vs* HBOT *vs* SOC	43.35 (PDGF), 43.8 (HBOT)	45	10 weeks	10 weeks	IAET stage III and IV	①②
Ma 2015	USA	23/23	46/0	PDGF *vs* SOC	59.3	60.1	40 weeks	16 weeks	Wagner: 1	①
Samuel 2016	India	29	18/11	PDGF *vs* SOC	56.09	—	24 weeks	24 weeks	Wagner: 1-3	①
Steed 1995	USA	61/57	89/29	PDGF *vs* SOC	63.2	58.3	20 weeks	until complete ulcer closure or up to 20 weeks	—	①
Wieman 1998	USA	255/127	255/127	PDGF *vs* SOC	58		32 weeks	20 weeks	IAET stage III and IV	①
Afshari 2005	Iran	30/20	27/23	EGF *vs* SOC	56.9	59.7	8 weeks	4 weeks	Wagner: 1–5	①②
Fern’andez-Montequ’ın 2009	Cuba	101/48	76/73	EGF *vs* SOC	65.2	64.0	1 year	8 weeks	Wagner: 3–4	①④
Gomez-Villa 2014	Mexico	17/17	21/13	EGF *vs* SOC	62.1	55.1	8 weeks	8 weeks	TEXAS: A/B	①
Oliveira 2021	Brazil	14/11	18/7	EGF *vs* SOC	60.6	65.1	12 weeks	12 weeks	—	①
Park 2018	South Korea	82/85	104/63	EGF *vs* SOC	56.52	59.31	12 weeks	12 weeks	Wagner: 1–2	①
Singla 2014	India	25/25	44/6	EGF *vs* SOC	58.80	55.84	8 weeks	8 weeks	Wagner: 1–2	①
Tsang 2003	China	42/19	29/32	EGF *vs* SOC	65.5	64.37	24 weeks	12 weeks	Wagner: 1–2	①④
Viswanathan 2020	India	27/23	27/23	EGF *vs* SOC	57.9	55.0	30 days	30 days	Wagner: 1–2	①②
Xu 2018	China	50/49	50/49	EGF *vs* SOC	65	63	8 weeks	8 weeks	Wagner: 2	②
Zhang 2021	China	40/40	38/42	EGF *vs* SOC	59	60	4 weeks	4 weeks	Wagner: 2–3	②
Jeppesen 2016	Denmark	11/12	16/7	ESWT *vs* SOC	65.3	67.8	7 weeks	3 weeks	Wagner: 1–2	③
Moretti 2009	Italy	15/15	16/14	ESWT *vs* SOC	56.8		20 weeks	6 days	—	①②
Nossair 2013	Egypt	20/20	29/11	ESWT *vs* SOC	56.6	55.15	12 weeks	3 weeks	Wagner: 2–3	③
Omar 2014	Saudi Arabia	19/19	27/11	ESWT *vs* SOC	56.59	57.0	20 weeks	8weeks	TEXAS: 1A/2A	①②③
Snyder 2018	USA	172/164	269/67	ESWT *vs* SOC	59.9	56.4	24 weeks	12 weeks	TEXAS: 1A/2A	①
Vangaveti 2023	Australia	25/23	34/14	ESWT *vs* SOC	62	62	6 weeks	6 weeks	TEXAS: 1A or higher	①
Wang 2009	China	34/36	—	ESWT *vs* HBOT	58.6	63.4	6 weeks	6 weeks	—	①
Wang 2011	China	39/38	—	ESWT *vs* HBOT	60.51	62.45	6 weeks	4 weeks	Wagner: 2–4	①
Ennis 2005	USA	27/28	—	LFU *vs* SOC	—		12 weeks	12 weeks	Wagner: 1–2	①②
Abd El Fattah 2018	Egypt	23/23	24/22	LFU *vs* SOC	32- 58	35- 67	12 weeks	12 weeks	TEXAS: 1–2A, 1–3B	①③
Lázaro Martínez 2020	Spain	27/24	48/3	LFU *vs* SOC	64.1	58	24 weeks	6 weeks	TEXAS: 1–2B/D	①②
Michailidis 2018	Australia	10	—	LFU *vs* SOC	—		24 weeks	24 weeks	TEXAS: 1–2A/B/C	①②
Rastogi 2019	India	34/26	—	LFU *vs* SOC	52.5	51.2	4 weeks	4 weeks	Wagner: 2–3	①③
Baker 1997	USA	41/39	55/25	ES *vs*SOC	54.1	51.5	4 weeks	4 weeks	—	①
Lundeberg 1992	Sweden	32/32	26/36	ES *vs* SOC	67.5	66	12 weeks	12 weeks	—	①
Peters 2001	USA	20/20	—	ES *vs* SOC	54.4	59.9	12 weeks	12 weeks	TEXAS: 1A/2A	①
Malekpour Alamdari 2021	Iran	30/30	33/27	NPWT *vs* SOC	70.31	71.80	12 weeks	—	Wagner: 2	④
Anjum 2022	Pakistan	20/20	28/12	NPWT *vs* SOC	42.95	46.30	10 weeks	8 weeks	Wagner: 1–2	①②
Blume 2008	USA	169/166	263/72	NPWT *vs* SOC	58	59	36 weeks	16 weeks	Wagner: 2–3	①④
Karatepe 2011	Turkey	30/37	19/48	NPWT *vs* SOC	68.5	66.3	—	—	—	②
Maranna 2021	India	22/23	33/12	NPWT *vs* SOC	50.23	49.00	12 weeks	2 weeks	Wagner: 1–2	①②
Nain 2011	India	15/15	25/5	NPWT *vs* SOC	61.33	55.40	8 weeks	8 weeks	—	①
Seidel 2020	Germany	171/174	267/78	NPWT *vs* SOC	67.6	68.1	24 weeks	16 weeks	Wagner: 1-3	①④
Seidel 2022	Germany	44/110	113/41	NPWT *vs* SOC	66.5	67.8	24 weeks	16 weeks	Wagner: 2–4	①②④
Ahmed 2017	Egypt	28/28	38/18	PRP *vs* SOC	43.2	49.8	12 weeks	12 weeks	TEXAS: 1–2A/C	①
Malekpour Alamdari 2021	Iran	43/47	56/34	PRP *vs* SOC	56.3	56.7	24 weeks	3 weeks	Wagner: 1–2	②
Driver 2006	USA	40/32	59/13	PRP *vs* SOC	—		24 weeks	12 weeks	TEXAS: 1A	①②
Elsaid 2020	Egypt	12/12	14/10	PRP *vs* SOC	54.7	55.6	20 weeks	20 weeks	—	①②
Gowsick 2023	India	87/87	104/70	PRP *vs* SOC	—		12 weeks	12 weeks	—	①③
Gupta 2021	India	30/30	41/19	PRP *vs* SOC	56.03	55.76	6 weeks	6 weeks	Wagner: 1–2	①③
Hossam 2022	Egypt	40/40	62/18	PRP *vs* SOC	54.9	54.8	12 weeks	12 weeks	Wagner: 1–2	①④
Li 2015	China	59/58	75/42	PRP *vs* SOC	61.4	64.1	12 weeks	12 weeks	—	①
Orban 2022	Egypt	36/36	41/31	PRP *vs* SOC	56.03	58.69	20 weeks	20 weeks	—	①②
Singh 2018	Etawah	29/26	34/21	PRP *vs* SOC	53.76	55.69	4 weeks	4 weeks	—	①②④
Xie 2020	China	25/23	27/21	PRP *vs* SOC	60.50	61.10	8 weeks	8 weeks	—	①②
Chen 2017	China	20/18	21/17	HBOT *vs* SOC	64.3	60.8	6 weeks	4 weeks	Wagner: 1-3	①④
Faglia 1996	Italy	35/33	48/20	HBOT *vs* SOC	61.7	65.6	—	Mean session = 38.8 ± 8	Wagner: 2–4	④
Abidia 2003	UK	9/9	9/9	HBOT *vs* SOC	72	70	1 year	6 weeks	Wagner: 1–2	①④
Duzgun 2008	Turkey	50/50	64/36	HBOT *vs* SOC	58.1	63.3	92 ± 12 weeks	6 weeks	Wagner: 2–4	①④
Fedorko 2016	Canada	49/54	69/34	HBOT *vs* SOC	61	62	12 weeks	6 weeks	Wagner: 2–4	①③④
Kessler 2003	France	14/13	19/8	HBOT *vs* SOC	60.2	67.6	6 weeks	2 weeks	Wagner: 1-3	①③
Kumar 2020	India	28/26	39/15	HBOT *vs* SOC	58.4	56.9	1 year	6 weeks	Wagner: 2–4	①④
Londahl 2010	Sweden	49/45	56/38	HBOT *vs* SOC	69	68	1 year	8 weeks	Wagner: 2–4	①④
Salama 2019	Egypt	15/15	22/8	HBOT *vs* SOC	55.1	57.7	16 weeks	4-8 weeks	Wagner: 2–3	①④
Santema 2018	Netherlands	60/60	97/23	HBOT *vs* SOC	67.6	70.6	1 year	8 weeks	Wagner: 2–4	①④
Al-Jalodi 2022	USA	81/64	—	TOT *vs* SOC	—		1 year	12 weeks	ISDA: 1 -2	①④
Driver 2017	USA	65/63	95/33	TOT *vs* SOC	59		12 weeks	12 weeks	TEXAS: 1A	①
Frykberg 2020	UK	36/37	63/10	TOT *vs* SOC	64.6	61.9	1 year	12 weeks	TEXAS: 1–2	①②④
He 2021	China	40/40	49/31	TOT *vs* SOC	62.7	63.1	1 year	8 weeks	Wagner: 2–4	①②③④
Niederauer 2015	USA	21/21	33/9	TOT *vs* SOC	58.3	59.2	12 weeks	12 weeks	—	①
Niederauer 2017	USA	50/50	79/21	TOT *vs* SOC	57.5	59.1	12 weeks	12 weeks	—	①
Niederauer 2018	USA	74/72	113/33	TOT *vs* SOC	56.1	56.6	12 weeks	12 weeks	TEXAS: 1A	①
Serena 2021	UK	81/64	107/37	TOT *vs* SOC	64.20	62.69	12 weeks	12 weeks	Wagner: 1–2	①③
Yu 2016	Canada	10/10	17/3	TOT *vs* SOC	57	58	8 weeks	8 weeks	—	①
Didomenico 2018	USA	40/40	54/26	AMT *vs* SOC	60.1	61.0	12 weeks	12 weeks	—	①②
Game 2021	UK	15/16	25/6	AMT *vs* SOC	62.8	57	12 weeks	12 weeks	—	①④
Lavery 2014	USA	50/47	68/29	AMT *vs* SOC	55.5	55.1	12 weeks	12 weeks	—	①④
Mohajeri-Tehrani 2016	Iran	27/30	37/20	AMT *vs* SOC	55.44	60	6 weeks	6 weeks	Wagner: 2–4	①④
Serena 2019	USA	38/38	59/17	AMT *vs* SOC	59.2	59.6	16 weeks	12 weeks	Wagner: 1–2	①
Snyder 2016	USA	15/14	25/4	AMT *vs* SOC	57.9	58.6	6 weeks	6 weeks	Wagner: 1–2	①
Tettelbach 2019a	USA	54/56	80/30	AMT *vs* SOC	57.4	57.1	16 weeks	12 weeks	—	①
Tettelbach 2019b	USA	101/54	126/29	AMT *vs* SOC	58.3	56.3	16 weeks	12 weeks	—	①
Thompson 2019	USA	7/6	11/2	AMT *vs* SOC	58.50	55.17	16 weeks	12 weeks	TEXAS: 1	①
Zelen 2013	USA	13/12	16/9	AMT *vs* SOC	56.4	61.7	12 weeks	6 weeks	—	①②
Zelen 2015	USA	20/20	19/21	AMT *vs* SOC	63.2	62.2	12 weeks	6 weeks	—	①
Zelen 2016	USA	32/35	41/26	AMT *vs* SOC	63.3	60.6	12 weeks	6 weeks	—	①②

**Notes.**

① Wound healing rate; ② Wound healing time; ③ percentage area reduction (PAR); ④ Amputation.

Afshari et al., 2005; Fernández-Montequín et al., 2009; Gomez-Villa et al., 2014; Oliveira et al., 2021; Park et al., 2018; Singla et al., 2014; Tsang et al., 2003; Viswanathan, Juttada & Babu, 2020; Xu et al., 2018; Zhang et al., 2021a; Agrawal et al., 2009; Bhansali et al., 2009; Blume et al., 2011; Jaiswal et al., 2010; Khandelwal et al., 2013; Ma et al., 2015; Samuel et al., 2016; Steed, 1995; Wieman, Smiell & Su, 1998; Ahmed et al., 2017; Driver et al., 2006; Elsaid et al., 2020; Gowsick et al., 2023; Gupta et al., 2021; Hossam et al., 2022; Li et al., 2015; Malekpour Alamdari et al., 2021b; Orban et al., 2022; Singh et al., 2018; Xie et al., 2020; Lu et al., 2008; Han, Kim & Kim, 2010; Huang et al., 2005; Jain et al., 2011; Lu et al., 2011; Mohammadzadeh et al., 2013; Ozturk et al., 2012; Uzun et al., 2021; Abd El Fattah et al., 2018; Ennis et al., 2005; Lázaro-Martínez et al., 2020; Michailidis et al., 2018; Rastogi, Bhansali & Ramachandran, 2019; Anjum et al., 2022; Blume et al., 2008; Karatepe et al., 2011; Malekpour Alamdari et al., 2021a; Maranna et al., 2021; Nain et al., 2011; Seidel, Lefering & DiaFu study, 2022; Seidel et al., 2020; Haze et al., 2022; Kaviani et al., 2011; Minatel et al., 2009; De Alencar Fonseca Santos et al., 2018; Naidu et al., 2005; Baker et al., 1997; Lundeberg, Eriksson & Malm, 1992; Peters et al., 2001; Jeppesen et al., 2016; Moretti et al., 2009; Nossair, Eid & Salama, 2013; Omar et al., 2014; Sandoval Ortíz et al., 2014; Snyder et al., 2018; Vangaveti et al., 2023; Wang et al., 2009; Wang, Wu & Yang, 2011; DiDomenico et al., 2018; Game et al., 2021; Lavery et al., 2014; Mohajeri-Tehrani et al., 2016; Serena et al., 2020; Snyder et al., 2016; Tettelbach et al., 2019a; Tettelbach et al., 2019b; Thompson et al., 2019; Zelen et al., 2015; Zelen et al., 2013; Zelen et al., 2016; Abidia et al., 2003; Chen et al., 2017; Duzgun et al., 2008; Faglia et al., 1996; Fedorko et al., 2016; Kessler et al., 2003; Kumar et al., 2020; Londahl et al., 2010; Salama et al., 2019; Santema et al., 2018; Al-Jalodi et al., 2022; Driver et al., 2017; Frykberg et al., 2020; He et al., 2021; Niederauer, Michalek & Armstrong, 2015; Niederauer, Michalek & Armstrong, 2017; Niederauer et al., 2018; Serena et al., 2021; Yu et al., 2016.

### Quality of the included studies

**Figure 3:**
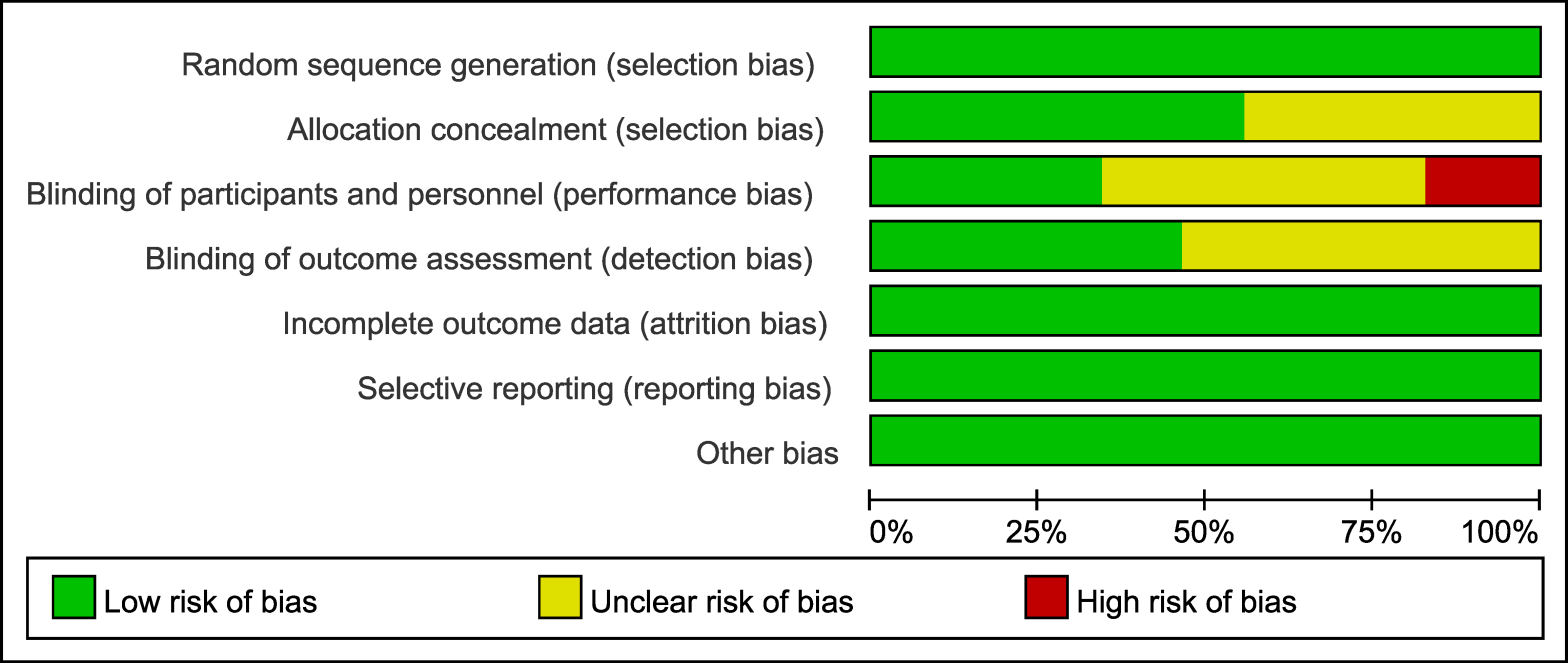
Risk of bias graph of the included studies. The proportion of studies rated as having low, unclear, or high risk of bias across seven domains is presented.

According to the Cochrane Collaboration standards, almost half of the studies failed to report the blinding of researchers, participants, and medical assessors, and the allocation concealment was not disclosed. 17 studies reported a high risk of bias because they were not blinded (Fig. 3, Supplemental Information 4).

### Wound healing rate

**Figure 4:**
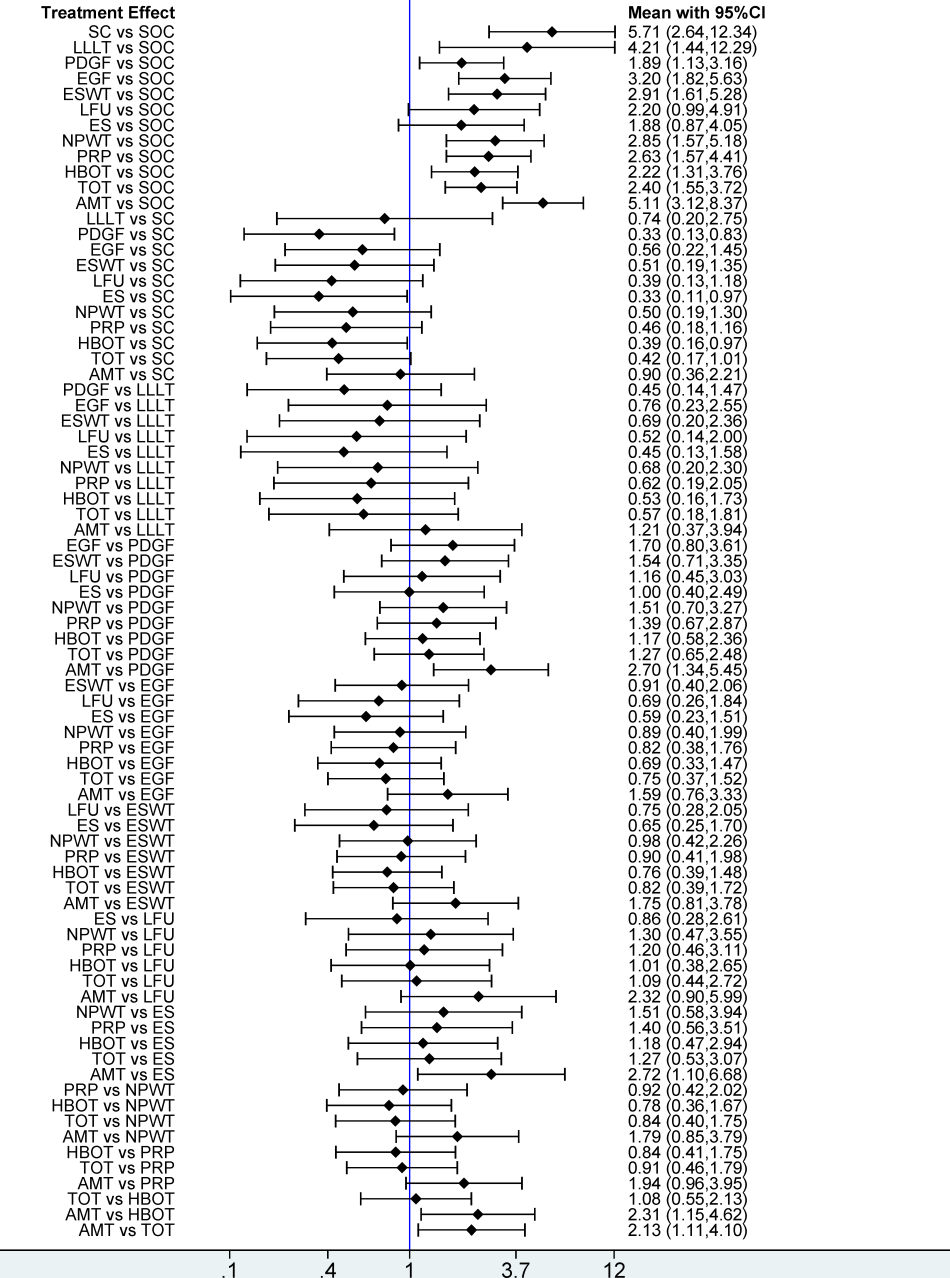
Forest plot of network meta-analysis results for wound healing rate. Treatment effects are presented as odds ratios (ORs) with 95% confidence intervals (CIs).

**Figure 5:**
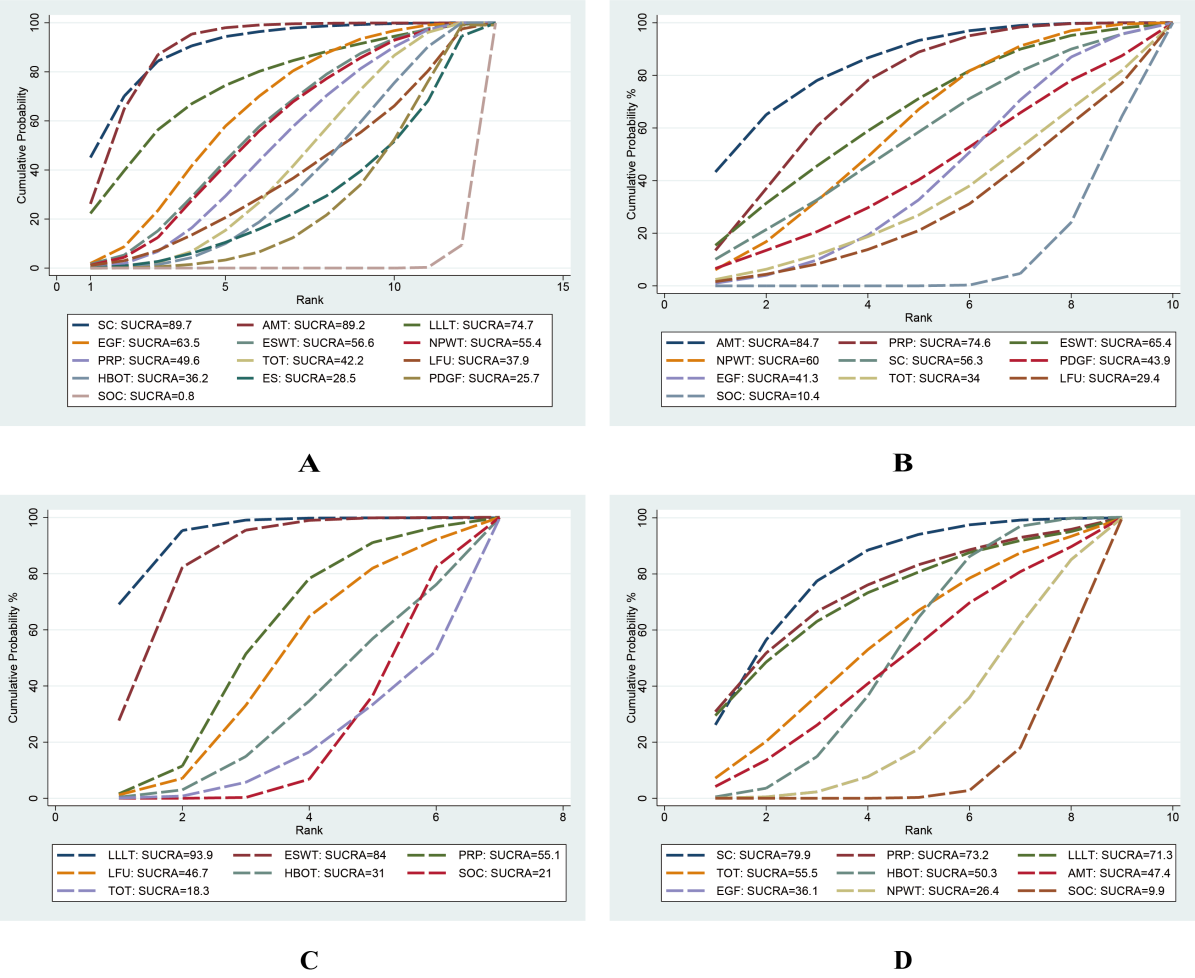
Surface Under the Cumulative Ranking (SUCRA) values of different interventions for four outcomes. (A) Wound healing rate; (B) wound healing time; (C) percentage area reduction (PAR); (D) amputation rate. Higher SUCRA values and larger areas under the curve indicate a greater probability that the intervention is more effective for the corresponding outcome.

This network meta-analysis evaluated 12 interventions for the treatment of DFU, emphasizing wound healing rate. Based on 95 RCTs involving 6,807 patients, our analysis revealed that, compared with SOC, only LFU (OR = 2.20; 95% CI [0.99–4.91]) and ES (OR = 1.88; 95% CI [0.87–4.05]) among the 12 evaluated interventions failed to achieve a statistically significant enhancement in wound healing rate. The remaining 10 interventions, including SC, AMT, LLLT, EGF, PDGF, ESWT, NPWT, PRP, HBOT, and TOT, significantly enhanced wound healing rate. In pairwise comparisons among the 12 interventions, SC significantly improved wound healing rate compared with PDGF (OR = 0.33; 95% CI [0.13–0.83]), ES (OR = 0.33; 95% CI [0.11–0.97]), and HBOT (OR = 0.39; 95% CI [0.16–0.97]). Additionally, AMT significantly outperformed PDGF (OR = 2.70; 95% CI [1.34–5.45]), ES (OR = 2.72; 95% CI [1.10–6.68]), HBOT (OR = 2.31; 95% CI [1.15–4.62]), and TOT (OR = 2.13; 95% CI [1.11–4.10]) in improving healing outcomes (see Fig. 4). According to the SUCRA rankings, SC (89.7%) and AMT (89.2%) ranked highest, while PDGF (25.7%) and SOC (0.8%) ranked lowest (see Fig. 5A). Supplemental Information 3 displays the ratios of different interventions for wound healing rate, with statistically significant findings highlighted in blue.

### Wound healing time

**Figure 6:**
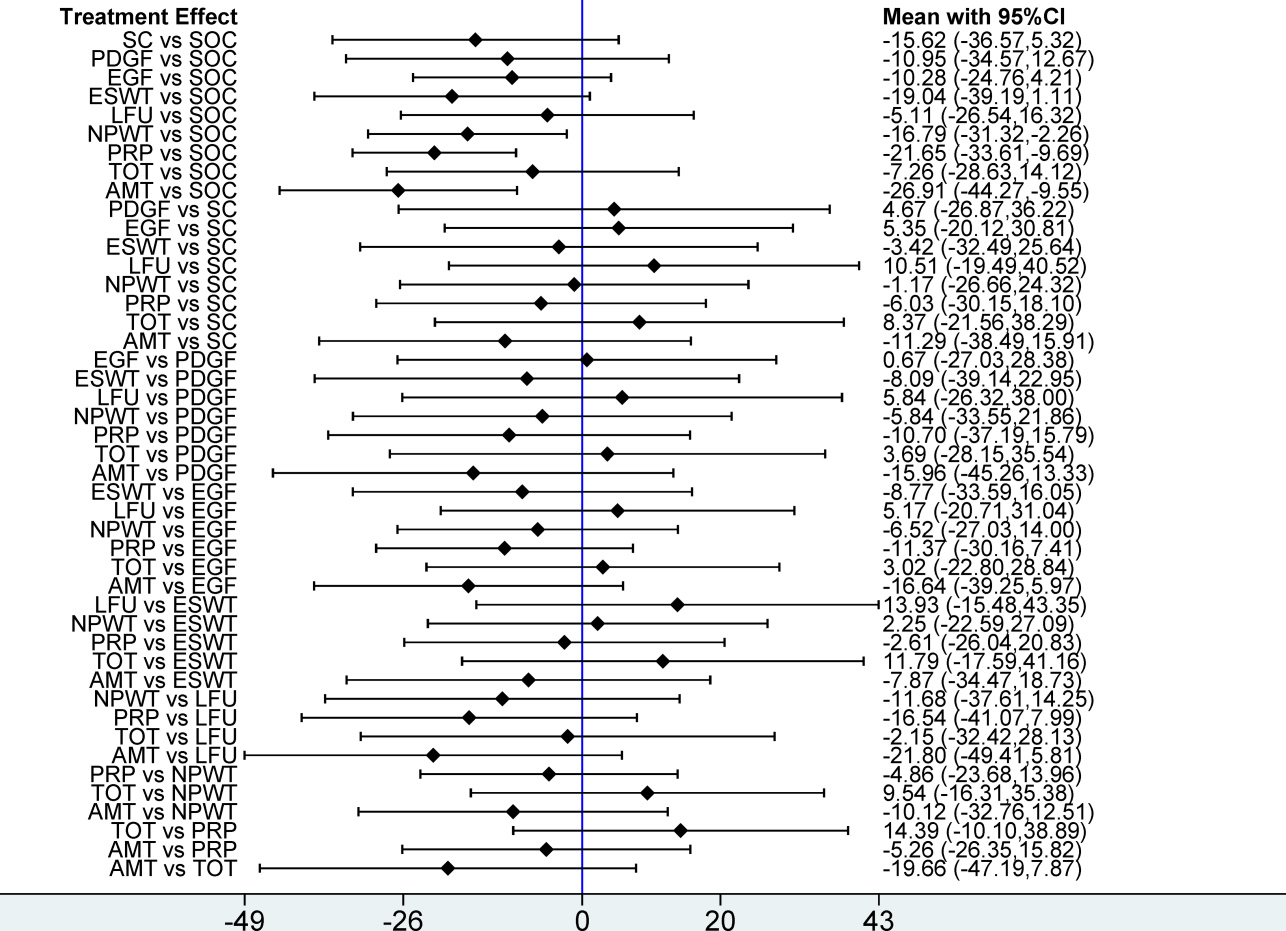
Forest plot of network meta-analysis results for wound healing time. Treatment effects are presented as mean differences (MDs) with 95% confidence intervals (CIs).

This network meta-analysis examined nine interventions for DFU: AMT, PRP, ESWT, NPWT, SC, PDGF, TOT, LFU, and EGF, primarily focusing on their mean healing times. Based on 28 RCTs involving 1,591 patients, our analysis demonstrated that, compared with SOC, AMT (MD = –26.91 days; 95% CI [−44.27 to −9.55]), PRP (MD = −21.65 days; 95% CI [−33.61 to −9.69]), and NPWT (MD = −16.79 days; 95% CI [−31.12 to −2.26]) significantly reduced woundr healing time. Among 10 interventions, AMT (SUCRA = 84.7%) and PRP (SUCRA = 74.6%) ranked highest according to SUCRA values, while LFU (29.4%) and SOC (10.4%) ranked lowest (see Figs. 5B, 6).

### PAR

**Figure 7:**
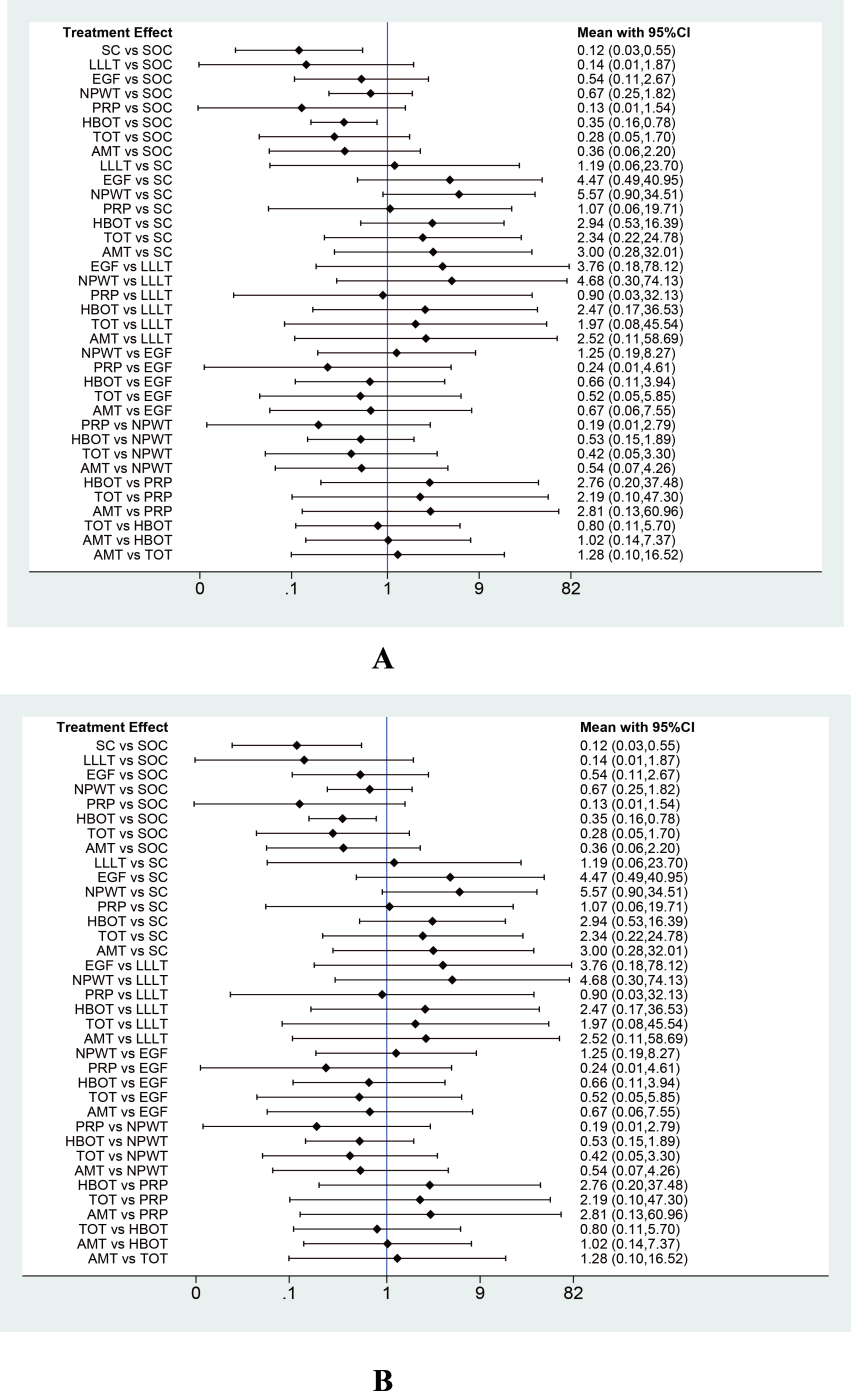
Forest plots of network meta-analysis results for (A) percentage area reduction (PAR) and (B) amputation rate. Treatment effects are presented as mean differences (MDs) for PAR and odds ratios (ORs) for amputation rate, with 95% confidence intervals (CIs).

This network meta-analysis evaluated six interventions for DFU: LLLT, ESWT, PRP, HBOT, LFU, and TOT, primarily focusing on PAR. Based on 15 RCTs involving 880 patients, our analysis revealed that, compared with SOC, LLLT (MD = 34.27; 95% CI: 17.35 to 51.20) and ESWT (MD = 27.50; 95% CI [11.00–44.00]) significantly reduced the PAR. Among the seven interventions evaluated, LLLT demonstrated a significantly greater reduction in ulcer area compared with both HBOT (MD = −31.42, 95% CI [−58.74 to −4.10]) and TOT (MD = −36.93, 95% CI [−63.54 to −10.31]). In addition, ESWT also significantly reduced PAR compared with TOT (MD = −30.15; 95% CI [−56.27 to −4.03]). According to the SUCRA rankings, LLLT (93.9%) and ESWT (84.0%) ranked highest, while SOC (21.0%) and TOT (18.3%) ranked lowest (see Figs. 5C, 7A).

### Amputation rate

This network meta-analysis evaluated eight interventions for DFU: PRP, LLLT, SC, TOT, HBOT, AMT, EGF, and NPWT, primarily focusing on amputation rate. Based on 30 RCTs involving 2,526 patients, our analysis revealed that, compared with SOC, SC (OR = 0.12; 95% CI [0.03–0.55]) and HBOT (OR = 0.35; 95% CI [0.16–0.78]) significantly reduced the amputation rate. According to the SUCRA rankings, SC (79.9%) and PRP (73.2%) ranked highest, whereas NPWT (26.4%) and SOC (9.9%) ranked lowest (refer to Figs. 5D, 7B).

### Subgroup analysis

**Figure 8:**
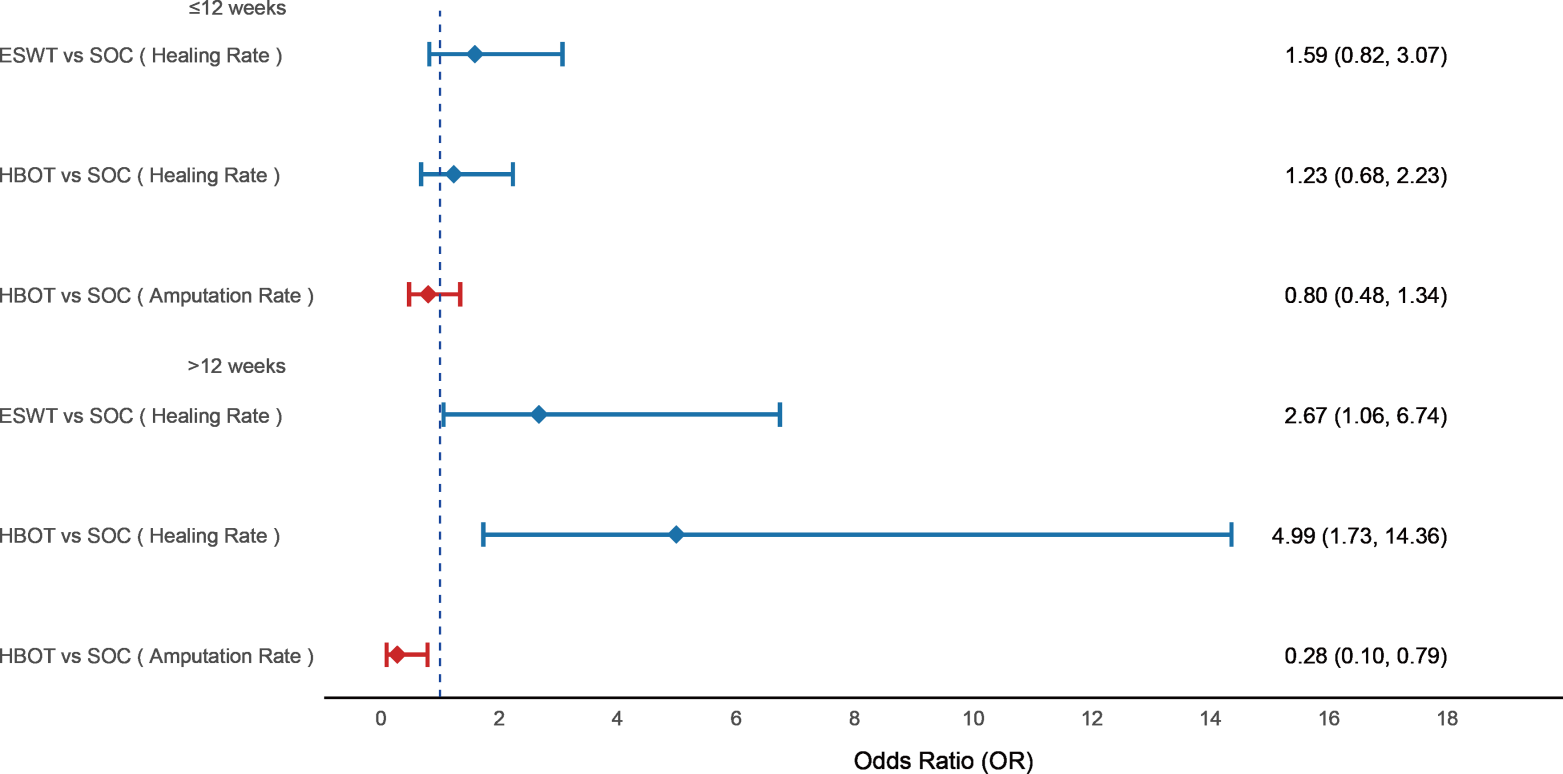
Forest plot of subgroup analysis based on follow-up duration. Subgroup 1 included studies with follow-up ≤ 12 weeks, and subgroup 2 included studies with follow-up >12 weeks. Treatment effects are presented as odds ratios (ORs) with 95% confidence intervals (CIs).

We performed a subgroup analysis based on follow-up duration: subgroup 1 included studies with follow-up ≤12 weeks, and subgroup 2 included those with follow-up >12 weeks. Regarding wound healing rate, neither ESWT nor HBOT showed a significant benefit compared to SOC within 12 weeks. However, beyond 12 weeks, both ESWT (OR = 2.67; 95% CI [1.06–6.74]) and HBOT (OR = 4.99; 95% CI [1.73–14.36]) significantly improved healing rate. For amputation rate, HBOT did not show a significant reduction within 12 weeks compared to SOC, but after more than 12 weeks, it significantly reduced the amputation rate (OR = 0.28; 95% CI [0.10–0.79]) (see Fig. 8).

### Inconsistency assessment

We evaluated inconsistency using global inconsistency tests and a node-splitting approach for all comparisons. The inconsistency tests indicated no significant inconsistency in this NMA (*p* = 0.2268). Outcomes from the node-splitting analysis, presented in Supplemental Information 3, indicate no inconsistency between direct and indirect comparisons.

### Small-scale study effect analysis

**Figure 9:**
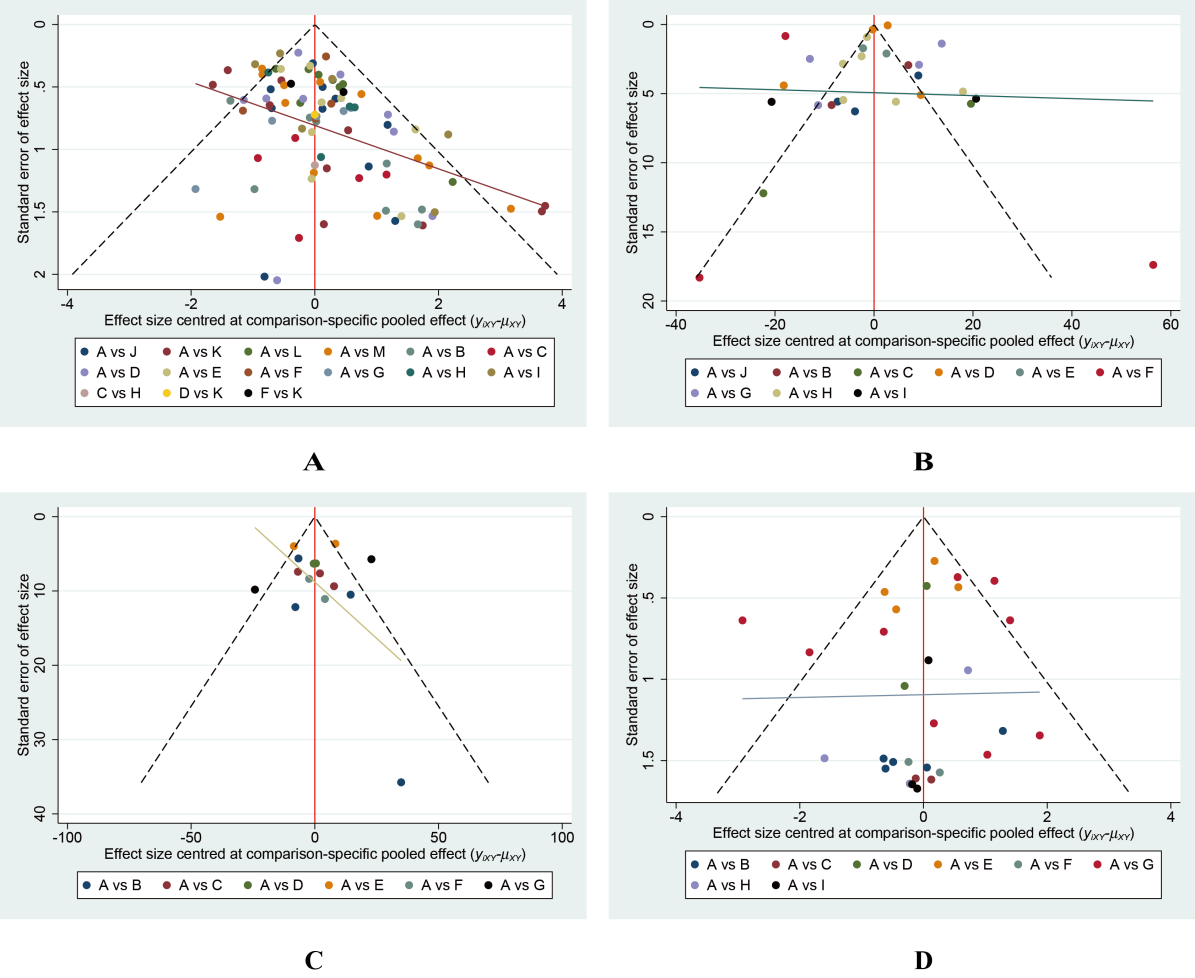
Funnel plots of the study treatments. (A) Wound healing rate; (B) Wound healing time; (C) Percentage area reduction (PAR); (D) Amputation rate.

Our analysis revealed no indication that smaller-scale studies influenced various outcomes (Fig. 9).

## Discussion

This study incorporated 99 RCTs involving 7,356 patients and utilized NMA to integrate direct and indirect comparisons (Rouse, Chaimani & Li, 2017), enhancing statistical power to systematically evaluate the effectiveness of 12 DFU treatments. Primary outcome measures included wound healing rate, wound healing time, PAR, and amputation rate. All interventions were compared against SOC or placebo. Our analysis showed that, compared to SOC, ten interventions—including SC, AMT, LLLT, EGF, PDGF, ESWT, NPWT, PRP, HBOT, and TOT—significantly improved wound healing rate. SC significantly outperformed PDGF, ES, and HBOT in promoting wound healing. Moreover, AMT was significantly more effective than PDGF, ES, HBOT, and TOT, with SC ranked highest according to SUCRA. In terms of wound healing time, AMT, PRP, and NPWT significantly reduced healing duration compared to SOC, with LLLT ranking highest in SUCRA. Regarding ulcer area reduction, both LLLT and ESWT were significantly superior to SOC. Furthermore, LLLT significantly outperformed HBOT and TOT, and ESWT also showed superiority over TOT, with LLLT achieving the highest SUCRA ranking. For amputation rate, SC and HBOT significantly reduced the risk compared to SOC, with SC ranked highest according to SUCRA.

In this NMA, we observed that multiple interventions demonstrated potential advantages in promoting DFU healing, particularly SC, AMT, and LLLT. Among these, the effect of SC was generally consistent with previous studies: multiple meta-analyses have confirmed that stem cells not only significantly improve wound healing rate and reduce the risk of amputation but also positively impact patients’ quality of life (Dai et al., 2020; Elsharkawi et al., 2023; Guo et al., 2017; Mudgal et al., 2024; Shu et al., 2018; Sun et al., 2022c; Zhang, Deng & Tang, 2017). Compared with SOC, SC demonstrated superior clinical performance in this study. Notably, our study is the first to reveal that SC was more effective than PDGF and HBOT in improving wound healing rate, which may be attributed to the multiple biological mechanisms of stem cells. In contrast, PDGF and HBOT exhibit certain limitations. PDGF promotes fibroblast proliferation and collagen synthesis, but its short half-life and rapid degradation limit sustained activity, and its isolated signaling lacks the broad paracrine effects of stem cells (Sun et al., 2024; Zhang et al., 2021b). HBOT enhances angiogenesis by increasing oxygen availability, but its indirect effects depend on patient-specific factors, and it lacks the multilineage differentiation and immunomodulatory capabilities of stem cells (Goldstein, 2013; Peña-Villalobos et al., 2018). The superior performance of SC may be explained by its comprehensive mechanisms, including the release of angiogenic growth factors, modulation of inflammation and immune responses, reduction of fibrosis, and optimization of the wound microenvironment through extracellular matrix remodeling (Panda & Nayak, 2024). Owing to their pluripotency and diverse paracrine and chemotactic effects, stem cells exhibit robust tissue regeneration potential both locally and systemically. Two recent NMAs involving SC compared its efficacy with that of PRP and EGF and found no significant differences (Yang et al., 2025). Another NMA included 11 different interventions and highlighted the superiority of SC over SOC, but did not find SC to be superior to other interventions or effective in reducing the risk of amputation (OuYang et al., 2024). These differences may be attributed to the scope of included studies, patient characteristics, follow-up duration, and stem cell types. The stem cell therapies included in this study primarily involve bone marrow-derived stem cells, adipose-derived stem cells, and peripheral blood-derived stem cells. Stem cells commonly used to treat DFU in clinical practice are categorized into two types: somatic stem cells and pluripotent stem cells. Among these, mesenchymal stem cells (MSCs) are the most commonly used form of somatic stem cells, with bone marrow-derived MSCs being the preferred cell type for clinical applications in diabetic wounds. Compared with bone marrow-derived MSCs, adipose-derived MSCs exhibit similar potential to enhance wound healing, and clinical research on peripheral blood stem cells is also increasing (Ho et al., 2023).

Second, consistent with previous studies, we found that AMT was significantly superior to SOC in improving wound healing rate and shortening wound healing time. Several meta-analyses demonstrated its effectiveness in significantly improving DFU healing rate and markedly reducing healing time (Haugh et al., 2017; Huang et al., 2020a; Laurent et al., 2017; Mohammed et al., 2022; Su et al., 2020). We also found that AMT was significantly superior to PDGF, ES, HBOT, and TOT in improving wound healing rate, potentially representing the first time this conclusion has been reported. A previous NMA of nine different dressings also demonstrated that amniotic membrane dressings had the greatest advantage in healing rate among multiple interventions (Zhang, Sun & Jiang, 2019). AMT’s therapeutic mechanisms involve abundant growth factors and key cellular components present in amniotic membranes, offering anti-inflammatory, anti-fibrotic, and angiogenesis-promoting effects. As a natural biological scaffold, AMT facilitates tissue regeneration and cell proliferation, especially beneficial in clinical scenarios requiring rapid wound closure (Koob et al., 2013; Munoz-Torres et al., 2022; Xiao et al., 2021). Third, we observed that LLLT was significantly superior to SOC in improving wound healing rate and reducing wound area, consistent with the findings of existing meta-analyses (Huang et al., 2021; Li et al., 2018; Santos et al., 2021; Zhou et al., 2021). Notably, this study found that LLLT was also superior to HBOT and TOT in reducing ulcer area. Currently, no NMA has systematically compared the efficacy of LLLT with multiple other interventions. LLLT promotes wound healing through photobiomodulation, significantly enhancing cellular metabolism, improving microcirculation, and stimulating collagen synthesis to expedite wound repair (Frangez et al., 2017; Peplow & Baxter, 2012; Tatmatsu-Rocha et al., 2016).

Subgroup analysis revealed that HBOT had no significant effect on healing rate and amputation rate within 12 weeks but demonstrated significant therapeutic effects after 12 weeks. This finding is consistent with previous observations suggesting the long-lasting effectiveness of HBOT in certain pathophysiological conditions involving irradiated tissues (Marx et al., 1990). One RCT also failed to detect significant differences at 12 weeks between HBOT and SOC (Fedorko et al., 2016). HBOT enhances tissue oxygenation, promotes collagen synthesis and angiogenesis, significantly reducing amputation risk (Huang et al., 2020b; Zhang et al., 2022). Similarly, ESWT did not demonstrate a healing advantage in the short term but significantly improved healing rate in follow-up periods longer than 12 weeks. One RCT found no significant difference in wound healing between the ESWT group and SOC during the first 12 weeks; however, a statistically significant difference emerged at week 20 (Snyder et al., 2018). ESWT accelerates tissue regeneration and local angiogenesis *via* mechanical stimulation (Cao et al., 2025). These findings suggest that the therapeutic effects of HBOT and ESWT are time-dependent, with cumulative benefits emerging after prolonged treatment, particularly beyond 12 weeks. However, no meta-analysis has yet conducted subgroup analyses of HBOT and ESWT based on follow-up duration, and this research gap warrants further attention.

Clarifying the specific advantages and clinical contexts of different therapies may assist clinicians in making personalized and staged treatment decisions. For patients who do not respond to SOC, priority should be given to SC, AMT, or LLLT. In particular, SC and AMT are more suitable for severe or refractory DFUs, such as neuroischemic ulcers, and can rapidly improve the wound surface in the early stages, whereas LLLT is better suited for outpatient clinics or resource-limited settings due to its high safety profile, relatively low cost, and ability to accelerate wound healing. Additionally, HBOT and ESWT demonstrate greater effectiveness in follow-up periods exceeding 12 weeks. HBOT can provide effective microcirculatory improvement for chronic ischemic ulcers, whereas ESWT is beneficial for patients with more severe fibrosis. However, advanced technologies such as SC, AMT, and HBOT require substantial investment in cost, equipment, and patient compliance, and their clinical application must be tailored to available medical resources and individual patient conditions to support comprehensive decision-making.

### Study limitation

The main limitation of this study is the lack of detailed classification of ulcer types. Most current RCTs do not provide detailed distinctions between different types of DFU (*e.g.*, neuropathic, ischemic, or mixed) in their reports, thereby precluding subgroup comparisons based on ulcer type. Future clinical studies should improve the reporting of DFU types and conduct more detailed population stratification analyses to provide more precise evidence-based support for individualized treatment strategies.

## Conclusion

In this NMA, we assessed 12 interventions for DFU, focusing on outcomes such as healing rate, wound healing times, PAR, and amputation rate. SC and AMT emerged as highly effective, significantly improving healing rate compared to PDGF, ES, and HBOT. SC was also associated with reduced amputation rate, while AMT significantly shortened wound healing time. LLLT exhibited considerable effectiveness in reducing ulcer areas. The therapeutic benefits of HBOT and ESWT appeared to be time-dependent, with greater effectiveness observed after 12 weeks. These results support a more individualized approach in treating DFU, where therapy selection can be customized based on specific treatment efficacies.

## Supplemental Information

10.7717/peerj.19809/supp-1 Supplemental Information 1 PRISMA checklist

10.7717/peerj.19809/supp-2 Supplemental Information 2 Search Strategy

10.7717/peerj.19809/supp-3 Supplemental Information 3 The ulcer healing rate

10.7717/peerj.19809/supp-4 Supplemental Information 4 Risk of bias summary of the included studies

10.7717/peerj.19809/supp-5 Supplemental Information 5 Original data

## Additional Information and Declarations

### Data Availability

The following information was supplied regarding data availability: The raw data is available in the Supplemental File.
