# Supplementary material for: Comparison of the efficacy of 12 interventions in the treatment of diabetic foot ulcers: a network meta-analysis
Source: PeerJ. 2025 Aug 11;13:e19809. doi: 10.7717/peerj.19809 (PMC12352421; doi:10.7717/peerj.19809)
Supplement: Supplemental Information 2 [file peerj-13-19809-s002.docx]

**Search Strategy**

**A systematic literature search was conducted to identify randomized controlled trials (RCTs) evaluating 13 intervention types for the treatment of diabetic foot ulcers (DFUs). The search was performed in PubMed, Web of Science, Cochrane Library, and Embase from their inception to September 2023.**

**The search strategy incorporated Medical Subject Headings (MeSH) terms and free-text terms, adapted to the syntax of each database. Filters were applied to include only RCTs, ensuring the selection of high-quality evidence.**

**The detailed search strategy for each database is provided below.**

**PubMed Search Strategy**

In PubMed, we used MeSH terms combined with free-text terms to retrieve relevant studies. The search terms included:

**((((Foot, Diabetic[Title/Abstract]) OR (Diabetic Feet[Title/Abstract])) OR (Feet, Diabetic[Title/Abstract])) OR (Foot Ulcer, Diabetic[Title/Abstract])) OR ("Diabetic Foot"[Mesh])**

**1.Epidermal growth factor**

**((((((((Growth Factor, Epidermal[Title/Abstract]) OR (Urogastrone[Title/Abstract])) OR (EGF[Title/Abstract])) OR (Human Urinary Gastric Inhibitor[Title/Abstract])) OR (beta-Urogastrone[Title/Abstract])) OR (beta Urogastrone[Title/Abstract])) OR (Epidermal Growth Factor-Urogastrone[Title/Abstract])) OR (Growth Factor-Urogastrone, Epidermal[Title/Abstract])) OR ("Epidermal Growth Factor"[Mesh])**

**2.Platelet-Derived Growth Factor**

**(((Factor, Platelet-Derived Growth[Title/Abstract]) OR (Growth Factor, Platelet-Derived[Title/Abstract])) OR (Platelet Derived Growth Factor[Title/Abstract])) OR ("Platelet-Derived Growth Factor"[Mesh])**

**3.Platelet-Rich Plasma**

**((Plasma, Platelet-Rich[Title/Abstract]) OR (Platelet Rich Plasma[Title/Abstract])) OR ("Platelet-Rich Plasma"[Mesh])**

**4. Stem Cells**

**(((((((((((((((Cell, Stem[Title/Abstract]) OR (Cells, Stem[Title/Abstract])) OR (Stem Cell[Title/Abstract])) OR (Progenitor Cells[Title/Abstract])) OR (Cell, Progenitor[Title/Abstract])) OR (Cells, Progenitor[Title/Abstract])) OR (Progenitor Cell[Title/Abstract])) OR (Mother Cells[Title/Abstract])) OR (Cell, Mother[Title/Abstract])) OR (Cells, Mother[Title/Abstract])) OR (Mother Cell[Title/Abstract])) OR (Colony-Forming Unit[Title/Abstract])) OR (Colony Forming Unit[Title/Abstract])) OR (Colony-Forming Units[Title/Abstract])) OR (Colony Forming Units[Title/Abstract])) OR ("Stem Cells"[Mesh])**

**5.**low‐frequency ultrasound

low‐frequency ultrasound [Title/Abstract]

6.Negative-Pressure Wound Therapy/ vacuum sealing drainage

("Negative-Pressure Wound Therapy"[Mesh]) OR ((((((((((((((((((((((((Negative Pressure Wound Therapy[Title/Abstract]) OR (Negative-Pressure Wound Therapies[Title/Abstract])) OR (Therapies, Negative-Pressure Wound[Title/Abstract])) OR (Therapy, Negative-Pressure Wound[Title/Abstract])) OR (Wound Therapies, Negative-Pressure[Title/Abstract])) OR (Wound Therapy, Negative-Pressure[Title/Abstract])) OR (Topical Negative-Pressure Therapy[Title/Abstract])) OR (Negative-Pressure Therapies, Topical[Title/Abstract])) OR (Negative-Pressure Therapy, Topical[Title/Abstract])) OR (Therapies, Topical Negative-Pressure[Title/Abstract])) OR (Therapy, Topical Negative-Pressure[Title/Abstract])) OR (Topical Negative Pressure Therapy[Title/Abstract])) OR (Topical Negative-Pressure Therapies[Title/Abstract])) OR (Negative-Pressure Dressings[Title/Abstract])) OR (Dressing, Negative-Pressure[Title/Abstract])) OR (Dressings, Negative-Pressure[Title/Abstract])) OR (Negative Pressure Dressings[Title/Abstract])) OR (Negative-Pressure Dressing[Title/Abstract])) OR (Vacuum-Assisted Closure[Title/Abstract])) OR (Closure, Vacuum-Assisted[Title/Abstract])) OR (Closures, Vacuum-Assisted[Title/Abstract])) OR (Vacuum Assisted Closure[Title/Abstract])) OR (Vacuum-Assisted Closures[Title/Abstract])) OR (vacuum sealing drainage[Title/Abstract]))

7. Low‐level laser therapy (LLLT)

((((((((((((((((((((((((((((((((Light Therapies, Low-Level[Title/Abstract]) OR (Light Therapy, Low-Level[Title/Abstract])) OR (Low Level Light Therapy[Title/Abstract])) OR (Low-Level Light Therapies[Title/Abstract])) OR (Therapies, Low-Level Light[Title/Abstract])) OR (Therapy, Low-Level Light[Title/Abstract])) OR (Photobiomodulation Therapy[Title/Abstract])) OR (Photobiomodulation Therapies[Title/Abstract])) OR (Therapies, Photobiomodulation[Title/Abstract])) OR (Therapy, Photobiomodulation[Title/Abstract])) OR (LLLT[Title/Abstract])) OR (Laser Therapy, Low-Level[Title/Abstract])) OR (Laser Therapies, Low-Level[Title/Abstract])) OR (Laser Therapy, Low Level[Title/Abstract])) OR (Low-Level Laser Therapies[Title/Abstract])) OR (Laser Irradiation, Low-Power[Title/Abstract])) OR (Irradiation, Low-Power Laser[Title/Abstract])) OR (Laser Irradiation, Low Power[Title/Abstract])) OR (Low-Power Laser Therapy[Title/Abstract])) OR (Low Power Laser Therapy[Title/Abstract])) OR (Laser Therapy, Low-Power[Title/Abstract])) OR (Laser Therapies, Low-Power[Title/Abstract])) OR (Laser Therapy, Low Power[Title/Abstract])) OR (Low-Power Laser Therapies[Title/Abstract])) OR (Low-Level Laser Therapy[Title/Abstract])) OR (Low Level Laser Therapy[Title/Abstract])) OR (Low-Power Laser Irradiation[Title/Abstract])) OR (Low Power Laser Irradiation[Title/Abstract])) OR (Laser Biostimulation[Title/Abstract])) OR (Biostimulation, Laser[Title/Abstract])) OR (Laser Phototherapy[Title/Abstract])) OR (Phototherapy, Laser[Title/Abstract])) OR ("Low-Level Light Therapy"[Mesh])

8. Electric Stimulation Therapy

**((((((((((((((Therapeutic Electrical Stimulation[Title/Abstract]) OR (Electrical Stimulation, Therapeutic[Title/Abstract])) OR (Stimulation, Therapeutic Electrical[Title/Abstract])) OR (Therapeutic Electric Stimulation[Title/Abstract])) OR (Electric Stimulation, Therapeutic[Title/Abstract])) OR (Stimulation, Therapeutic Electric[Title/Abstract])) OR (Electrical Stimulation Therapy[Title/Abstract])) OR (Stimulation Therapy, Electrical[Title/Abstract])) OR (Therapy, Electrical Stimulation[Title/Abstract])) OR (Therapy, Electric Stimulation[Title/Abstract])) OR (Stimulation Therapy, Electric[Title/Abstract])) OR (Electrotherapy[Title/Abstract])) OR (Interferential Current Electrotherapy[Title/Abstract])) OR (Electrotherapy, Interferential Current[Title/Abstract])) OR ("Electric Stimulation Therapy"[Mesh])**

9. Extracorporeal Shockwave Therapy

(((((((((((((((Extracorporeal Shockwave Therapies[Title/Abstract]) OR (Shockwave Therapies, Extracorporeal[Title/Abstract])) OR (Shockwave Therapy, Extracorporeal[Title/Abstract])) OR (Therapy, Extracorporeal Shockwave[Title/Abstract])) OR (Shock Wave Therapy[Title/Abstract])) OR (Shock Wave Therapies[Title/Abstract])) OR (Therapy, Shock Wave[Title/Abstract])) OR (Extracorporeal Shock Wave Therapy[Title/Abstract])) OR (Extracorporeal High-Intensity Focused Ultrasound Therapy[Title/Abstract])) OR (Extracorporeal High Intensity Focused Ultrasound Therapy[Title/Abstract])) OR (HIFU Therapy[Title/Abstract])) OR (HIFU Therapies[Title/Abstract])) OR (Therapy, HIFU[Title/Abstract])) OR (High-Intensity Focused Ultrasound Therapy[Title/Abstract])) OR (High Intensity Focused Ultrasound Therapy[Title/Abstract])) OR ("Extracorporeal Shockwave Therapy"[Mesh])

10. **Amnion** /Biological Dressings

**Amnion[MeSH] OR Chorion[MeSH] OR Biological Dressings[MeSH] OR placental membrane OR fetal membrane OR amniotic membrane OR amnion OR chorion OR Grafix OR EpiFix**

**11.** HBOT

("Hyperbaric Oxygenation"[Mesh]) OR ((((((((( (Hyperbaric Oxygenations[Title/Abstract])) OR (Oxygenations, Hyperbaric[Title/Abstract])) OR (Hyperbaric Oxygen Therapy[Title/Abstract])) OR (Hyperbaric Oxygen Therapies[Title/Abstract])) OR (Oxygen Therapies, Hyperbaric[Title/Abstract])) OR (Oxygen Therapy, Hyperbaric[Title/Abstract])) OR (Therapies, Hyperbaric Oxygen[Title/Abstract])) OR (Therapy, Hyperbaric Oxygen[Title/Abstract])) OR (Oxygenation, Hyperbaric[Title/Abstract]))

**12.** TOT

(((Topical Oxygen[Title/Abstract]) OR (Topical Wound Oxygen[Title/Abstract])) OR (Continuous Diffusion of Oxygen[Title/Abstract])) OR (Continuous Topical Oxygen[Title/Abstract])

13.ozone (Excluded later due to eligibility criteria update)

**(**ozone**[Title/Abstract]) OR (**ozon **[Title/Abstract])**

**Web of science**

In Web of Science, we performed a topic-based search (TS=) using a combination of MeSH terms and free-text terms to identify relevant studies. The search terms included:

((TS=(Diabetic Foot OR Foot, Diabetic OR Diabetic Feet OR Feet, Diabetic OR Foot Ulcer, Diabetic)))

1.Epidermal growth factor

TS= **(Growth Factor, Epidermal OR Urogastrone OR EGF OR Human Urinary Gastric Inhibitor OR beta-Urogastrone OR beta Urogastrone OR Epidermal Growth Factor-Urogastrone OR Growth Factor-Urogastrone, Epidermal OR Epidermal Growth Factor)**

2.Platelet-Derived Growth Factor

TS=( **Factor, Platelet-Derived Growth** OR **Growth Factor, Platelet-Derived** OR **Platelet Derived Growth Factor** OR **Platelet-Derived Growth Factor** )

3.Platelet-Rich Plasma

TS=( **Plasma, Platelet-Rich** OR **Platelet Rich Plasma** OR **Platelet-Rich Plasma**)

4. Stem Cells

TS=( **Cell, Stem** OR **Cells, Stem** OR **Stem Cell** OR **Progenitor Cells** OR **Cell, Progenitor** OR **Cells, Progenitor** OR **Progenitor Cell** OR **Mother Cells** OR **Cell, Mother** OR **Cells, Mother** OR **Mother Cell** OR **Colony-Forming Unit** OR **Colony Forming Unit** OR **Colony-Forming Units** OR **Colony Forming Units** OR **Stem Cells**)

5. low‐frequency ultrasound

TS=( low‐frequency ultrasound)

6. Negative-Pressure Wound Therapy/ vacuum sealing drainage

TS=( Negative-Pressure Wound Therapy OR Negative Pressure Wound Therapy OR Negative-Pressure Wound Therapies OR Therapies, Negative-Pressure Wound OR Therapy, Negative-Pressure Wound OR Wound Therapies, Negative-Pressure OR Wound Therapy, Negative-Pressure OR Topical Negative-Pressure Therapy OR Negative-Pressure Therapies, Topical OR Negative-Pressure Therapy, Topical OR Therapies, Topical Negative-Pressure OR Therapy, Topical Negative-Pressure OR Topical Negative Pressure Therapy OR Topical Negative-Pressure Therapies OR Negative-Pressure Dressings OR Dressing, Negative-Pressure OR Dressings, Negative-Pressure OR Negative Pressure Dressings OR Negative-Pressure Dressing OR Vacuum-Assisted Closure OR Closure, Vacuum-Assisted OR Closures, Vacuum-Assisted OR Vacuum Assisted Closure OR Vacuum-Assisted Closures OR vacuum sealing drainage)

7.Low‐level laser therapy (LLLT)

TS=( Light Therapies, Low-Level OR Light Therapy, Low-Level OR Low Level Light Therapy OR Low-Level Light Therapies OR Therapies, Low-Level Light OR Therapy, Low-Level Light OR Photobiomodulation Therapy OR Photobiomodulation Therapies OR Therapies, Photobiomodulation OR Therapy, Photobiomodulation OR LLLT OR Laser Therapy, Low-Level OR Laser Therapies, Low-Level OR Laser Therapy, Low Level OR Low-Level Laser Therapies OR Laser Irradiation, Low-Power OR Irradiation, Low-Power Laser OR Laser Irradiation, Low Power OR Low-Power Laser Therapy OR Low Power Laser Therapy OR Laser Therapy, Low-Power OR Laser Therapies, Low-Power OR Laser Therapy, Low Power OR Low-Power Laser Therapies OR Low-Level Laser Therapy OR Low Level Laser Therapy OR Low-Power Laser Irradiation OR Low Power Laser Irradiation OR Laser Biostimulation OR Biostimulation, Laser OR Laser Phototherapy OR Phototherapy, Laser OR Low-Level Light Therapy)

8. Electric Stimulation Therapy

TS=( **Therapeutic Electrical Stimulation** OR **Electrical Stimulation, Therapeutic** OR **Stimulation, Therapeutic Electrical** OR **Therapeutic Electric Stimulation** OR **Electric Stimulation, Therapeutic** OR **Stimulation, Therapeutic Electric** OR **Electrical Stimulation Therapy** OR **Stimulation Therapy, Electrical** OR **Therapy, Electrical Stimulation** OR **Therapy, Electric Stimulation** OR **Stimulation Therapy, Electric** OR **Electrotherapy** OR **Interferential Current Electrotherapy** OR **Electrotherapy, Interferential Current** OR **Electric Stimulation Therapy**)

9. Extracorporeal Shockwave Therapy

TS=( Extracorporeal Shockwave Therapies OR Shockwave Therapies, Extracorporeal OR Shockwave Therapy, Extracorporeal OR Therapy, Extracorporeal Shockwave OR Shock Wave Therapy OR Shock Wave Therapies OR Therapy, Shock Wave OR Extracorporeal Shock Wave Therapy OR Extracorporeal High-Intensity Focused Ultrasound Therapy OR Extracorporeal High Intensity Focused Ultrasound Therapy OR HIFU Therapy OR HIFU Therapies OR Therapy, HIFU OR High-Intensity Focused Ultrasound Therapy OR High Intensity Focused Ultrasound Therapy OR Extracorporeal Shockwave Therapy)

10.Amnion /Biological Dressings

TS=( **Amnion OR Chorion OR Biological Dressings OR placental membrane OR fetal membrane OR amniotic membrane OR amnion OR chorion OR Grafix OR EpiFix**)

11.HBOT

TS=(Hyperbaric Oxygenation ORHyperbaric Oxygenations OR Oxygenations, Hyperbaric OR Hyperbaric Oxygen Therapy OR Hyperbaric Oxygen Therapies OR Oxygen Therapies, Hyperbaric OR Oxygen Therapy, Hyperbaric OR Therapies, Hyperbaric Oxygen OR Therapy, Hyperbaric Oxygen OR Oxygenation, Hyperbaric)

12.TOT

TS =(Topical Oxygen OR Topical Wound Oxygen OR Continuous Diffusion of Oxygen OR Continuous Topical Oxygen)

13.ozone (Excluded later due to eligibility criteria update)

TS=(ozone OR ozon)

**Cochrane Library**

We searched Cochrane Library using MeSH terms (same as PubMed) and free-text terms in the title (ti), abstract (ab), and keyword (kw) fields to identify RCTs. The search included:

(Foot, Diabetic):ti,ab,kw OR (Diabetic Feet):ti,ab,kw OR (Feet, Diabetic):ti,ab,kw OR (Foot Ulcer, Diabetic):ti,ab,kw

**1.Epidermal growth factor**

(**Growth Factor, Epidermal**):ti,ab,kw OR (**Urogastrone**):ti,ab,kw OR (**EGF**):ti,ab,kw OR (**Human Urinary Gastric Inhibitor**):ti,ab,kw OR (**beta-Urogastrone**):ti,ab,kw OR (**beta Urogastrone**):ti,ab,kw OR (**Epidermal Growth Factor-Urogastrone**):ti,ab,kw OR (**Growth Factor-Urogastrone, Epidermal**):ti,ab,kw

**2.Platelet-Derived Growth Factor**

(**Factor, Platelet-Derived Growth**):ti,ab,kw OR (**Growth Factor, Platelet-Derived**):ti,ab,kw OR (**Platelet Derived Growth Factor**):ti,ab,kw

**3.Platelet-Rich Plasma**

(**Plasma, Platelet-Rich**):ti,ab,kw OR (**Platelet Rich Plasma**):ti,ab,kw

**4. Stem Cells**

(**Cell, Stem**):ti,ab,kw OR (**Cells, Stem**):ti,ab,kw OR (**Stem Cell**):ti,ab,kw OR (**Progenitor Cells**):ti,ab,kw OR (**Cell, Progenitor**):ti,ab,kw OR (**Cells, Progenitor**):ti,ab,kw OR (**Progenitor Cell**):ti,ab,kw OR (**Mother Cells**):ti,ab,kw OR (**Cell, Mother**):ti,ab,kw OR (**Cells, Mother**):ti,ab,kw OR (**Mother Cell**):ti,ab,kw OR (**Colony-Forming Unit**):ti,ab,kw OR (**Colony Forming Unit**):ti,ab,kw OR (**Colony-Forming Units**):ti,ab,kw OR (**Colony Forming Units**):ti,ab,kw

**5.**low‐frequency ultrasound

(low‐frequency ultrasound):ti,ab,kw

6.Negative-Pressure Wound Therapy/ vacuum sealing drainage

(Negative Pressure Wound Therapy):ti,ab,kw OR (Negative-Pressure Wound Therapies):ti,ab,kw OR (Therapies, Negative-Pressure Wound):ti,ab,kw OR (Therapy, Negative-Pressure Wound):ti,ab,kw OR (Wound Therapies, Negative-Pressure):ti,ab,kw OR (Wound Therapy, Negative-Pressure):ti,ab,kw OR (Topical Negative-Pressure Therapy):ti,ab,kw OR (Negative-Pressure Therapies, Topical):ti,ab,kw OR (Negative-Pressure Therapy, Topical):ti,ab,kw OR (Therapies, Topical Negative-Pressure):ti,ab,kw OR (Therapy, Topical Negative-Pressure):ti,ab,kw OR (Topical Negative Pressure Therapy):ti,ab,kw OR (Topical Negative-Pressure Therapies):ti,ab,kw OR (Negative-Pressure Dressings):ti,ab,kw OR (Dressing, Negative-Pressure):ti,ab,kw OR (Dressings, Negative-Pressure):ti,ab,kw OR (Negative Pressure Dressings):ti,ab,kw OR (Negative-Pressure Dressing):ti,ab,kw OR (Vacuum-Assisted Closure):ti,ab,kw OR (Closure, Vacuum-Assisted):ti,ab,kw OR (Closures, Vacuum-Assisted):ti,ab,kw OR (Vacuum Assisted Closure):ti,ab,kw OR (Vacuum-Assisted Closures):ti,ab,kw OR (vacuum sealing drainage):ti,ab,kw

7. Low‐level laser therapy (LLLT)

(Light Therapies, Low-Level):ti,ab,kw OR (Light Therapy, Low-Level):ti,ab,kw OR (Low Level Light Therapy):ti,ab,kw OR (Low-Level Light Therapies):ti,ab,kw OR (Therapies, Low-Level Light):ti,ab,kw OR (Therapy, Low-Level Light):ti,ab,kw OR (Photobiomodulation Therapy):ti,ab,kw OR (Photobiomodulation Therapies):ti,ab,kw OR (Therapies, Photobiomodulation):ti,ab,kw OR (Therapy, Photobiomodulation):ti,ab,kw OR (LLLT):ti,ab,kw OR (Laser Therapy, Low-Level):ti,ab,kw OR (Laser Therapies, Low-Level):ti,ab,kw OR (Laser Therapy, Low Level):ti,ab,kw OR (Low-Level Laser Therapies):ti,ab,kw OR (Laser Irradiation, Low-Power):ti,ab,kw OR (Irradiation, Low-Power Laser):ti,ab,kw OR (Laser Irradiation, Low Power):ti,ab,kw OR (Low-Power Laser Therapy):ti,ab,kw OR (Low Power Laser Therapy):ti,ab,kw OR (Laser Therapy, Low-Power):ti,ab,kw OR (Laser Therapies, Low-Power):ti,ab,kw OR (Laser Therapy, Low Power):ti,ab,kw OR (Low-Power Laser Therapies):ti,ab,kw OR (Low-Level Laser Therapy):ti,ab,kw OR (Low Level Laser Therapy):ti,ab,kw OR (Low-Power Laser Irradiation):ti,ab,kw OR (Low Power Laser Irradiation):ti,ab,kw OR (Laser Biostimulation):ti,ab,kw OR (Biostimulation, Laser):ti,ab,kw OR (Laser Phototherapy):ti,ab,kw OR (Phototherapy, Laser):ti,ab,kw

8. Electric Stimulation Therapy

(**Therapeutic Electrical Stimulation**):ti,ab,kw OR (**Electrical Stimulation, Therapeutic**):ti,ab,kw OR (**Stimulation, Therapeutic Electrical**):ti,ab,kw OR (**Therapeutic Electric Stimulation**):ti,ab,kw OR (**Electric Stimulation, Therapeutic**):ti,ab,kw OR (**Stimulation, Therapeutic Electric**):ti,ab,kw OR (**Electrical Stimulation Therapy**):ti,ab,kw OR (**Stimulation Therapy, Electrical**):ti,ab,kw OR (**Therapy, Electrical Stimulation**):ti,ab,kw OR (**Therapy, Electric Stimulation**):ti,ab,kw OR (**Stimulation Therapy, Electric**):ti,ab,kw OR (**Electrotherapy**):ti,ab,kw OR (**Interferential Current Electrotherapy**):ti,ab,kw OR (**Electrotherapy, Interferential Current**):ti,ab,kw

9. Extracorporeal Shockwave Therapy

(Extracorporeal Shockwave Therapies):ti,ab,kw OR (Shockwave Therapies, Extracorporeal):ti,ab,kw OR (Shockwave Therapy, Extracorporeal):ti,ab,kw OR (Therapy, Extracorporeal Shockwave):ti,ab,kw OR (Shock Wave Therapy):ti,ab,kw OR (Shock Wave Therapies):ti,ab,kw OR (Therapy, Shock Wave):ti,ab,kw OR (Extracorporeal Shock Wave Therapy):ti,ab,kw OR (Extracorporeal High-Intensity Focused Ultrasound Therapy):ti,ab,kw OR (Extracorporeal High Intensity Focused Ultrasound Therapy):ti,ab,kw OR (HIFU Therapy):ti,ab,kw OR (HIFU Therapies):ti,ab,kw OR (Therapy, HIFU):ti,ab,kw OR (High-Intensity Focused Ultrasound Therapy):ti,ab,kw OR (High Intensity Focused Ultrasound Therapy):ti,ab,kw

10. **Amnion** /Biological Dressings

(**placental membrane**):ti,ab,kw OR (**fetal membrane**):ti,ab,kw OR (**amniotic membrane**):ti,ab,kw OR (**amnion**):ti,ab,kw OR (**chorion**):ti,ab,kw OR (**Grafix**):ti,ab,kw OR (**EpiFix**):ti,ab,kw

**11.** HBOT

(Hyperbaric Oxygenations):ti,ab,kw OR (Oxygenations, Hyperbaric):ti,ab,kw OR (Hyperbaric Oxygen Therapy):ti,ab,kw OR (Hyperbaric Oxygen Therapies):ti,ab,kw OR (Oxygen Therapies, Hyperbaric):ti,ab,kw OR (Oxygen Therapy, Hyperbaric):ti,ab,kw OR (Therapies, Hyperbaric Oxygen):ti,ab,kw OR (Therapy, Hyperbaric Oxygen):ti,ab,kw OR (Oxygenation, Hyperbaric):ti,ab,kw

12.TOT

(topical oxygen):ti,ab,kw OR (Topical Wound Oxygen):ti,ab,kw OR (Continuous Diffusion of Oxygen):ti,ab,kw OR (Continuous Topical Oxygen):ti,ab,kw

13. ozone (Excluded later due to eligibility criteria update)

(ozone):ti,ab,kw OR (ozon):ti,ab,kw

**Embase**

We searched Embase using Emtree terms (same as MeSH terms in PubMed) and free-text terms in the title (ti) and abstract (ab) fields to identify RCTs. The search included:

‘Foot, Diabetic’:ab,ti OR ‘Diabetic Feet’:ab,ti OR ‘Feet, Diabetic’:ab,ti OR ‘Foot Ulcer, Diabetic’:ab,ti

**1.Epidermal growth factor**

‘**Growth Factor, Epidermal**’:ab,ti OR ‘**Urogastrone**’:ab,ti OR ‘**Human Urinary Gastric Inhibitor**’:ab,ti OR ‘**beta-Urogastrone**’:ab,ti OR ‘**beta Urogastrone**’:ab,ti OR ‘**Epidermal Growth Factor-Urogastrone**’:ab,ti OR ‘**Growth Factor-Urogastrone, Epidermal**’:ab,ti

**2.Platelet-Derived Growth Factor**

‘**Factor, Platelet-Derived Growth**’:ab,ti OR ‘**Growth Factor, Platelet-Derived**’:ab,ti OR ‘**Platelet Derived Growth Factor**’:ab,ti

**3.Platelet-Rich Plasma**

‘**Plasma, Platelet-Rich**’:ab,ti OR ‘**Platelet Rich Plasma**’:ab,ti

**4. Stem Cells**

‘**Cell, Stem**’:ab,ti OR ‘**Cells, Stem**’:ab,ti OR ‘**Stem Cell**’:ab,ti OR ‘**Progenitor Cells**’:ab,ti OR ‘**Cell, Progenitor**’:ab,ti OR ‘**Cells, Progenitor**’:ab,ti OR ‘**Progenitor Cell**’:ab,ti OR ‘**Mother Cells**’:ab,ti OR ‘**Cell, Mother**’:ab,ti OR ‘**Cells, Mother**’:ab,ti OR ‘**Mother Cell**’:ab,ti OR ‘**Colony-Forming Unit**’:ab,ti OR ‘**Colony Forming Unit**’:ab,ti OR ‘**Colony-Forming Units**’:ab,ti OR ‘**Colony Forming Units**’:ab,ti

5.low‐frequency ultrasound

‘low‐frequency ultrasound’:ab,ti

6.Negative-Pressure Wound Therapy/ vacuum sealing drainage

‘Negative Pressure Wound Therapy’:ab,ti OR ‘Negative-Pressure Wound Therapies’:ab,ti OR ‘Therapies, Negative-Pressure Wound’:ab,ti OR ‘Therapy, Negative-Pressure Wound’:ab,ti OR ‘Wound Therapies, Negative-Pressure’:ab,ti OR ‘Wound Therapy, Negative-Pressure’:ab,ti OR ‘Topical Negative-Pressure Therapy’:ab,ti OR ‘Negative-Pressure Therapies, Topical’:ab,ti OR ‘Negative-Pressure Therapy, Topical’:ab,ti OR ‘Therapies, Topical Negative-Pressure’:ab,ti OR ‘Therapy, Topical Negative-Pressure’:ab,ti OR ‘Topical Negative Pressure Therapy’:ab,ti OR ‘Topical Negative-Pressure Therapies’:ab,ti OR ‘Negative-Pressure Dressings’:ab,ti OR ‘Dressing, Negative-Pressure’:ab,ti OR ‘Dressings, Negative-Pressure’:ab,ti OR ‘Negative Pressure Dressings’:ab,ti OR ‘Negative-Pressure Dressing’:ab,ti OR ‘Vacuum-Assisted Closure’:ab,ti OR ‘Closure, Vacuum-Assisted’:ab,ti OR ‘Closures, Vacuum-Assisted’:ab,ti OR ‘Vacuum Assisted Closure’:ab,ti OR ‘Vacuum-Assisted Closures’:ab,ti OR ‘vacuum sealing drainage’:ab,ti

7. Low‐level laser therapy (LLLT)

‘Light Therapies, Low-Level’:ab,ti OR ‘Light Therapy, Low-Level’:ab,ti OR ‘Low Level Light Therapy’:ab,ti OR ‘Low-Level Light Therapies’:ab,ti OR ‘Therapies, Low-Level Light’:ab,ti OR ‘Therapy, Low-Level Light’:ab,ti OR ‘Photobiomodulation Therapy’:ab,ti OR ‘Photobiomodulation Therapies’:ab,ti OR ‘Therapies, Photobiomodulation’:ab,ti OR ‘Therapy, Photobiomodulation’:ab,ti OR ‘LLLT’:ab,ti OR ‘Laser Therapy, Low-Level’:ab,ti OR ‘Laser Therapies, Low-Level’:ab,ti OR ‘Laser Therapy, Low Level’:ab,ti OR ‘Low-Level Laser Therapies’:ab,ti OR ‘Laser Irradiation, Low-Power’:ab,ti OR ‘Irradiation, Low-Power Laser’:ab,ti OR ‘Laser Irradiation, Low Power’:ab,ti OR ‘Low-Power Laser Therapy’:ab,ti OR ‘Low Power Laser Therapy’:ab,ti OR ‘Laser Therapy, Low-Power’:ab,ti OR ‘Laser Therapies, Low-Power’:ab,ti OR ‘Laser Therapy, Low Power’:ab,ti OR ‘Low-Power Laser Therapies’:ab,ti OR ‘Low-Level Laser Therapy’:ab,ti OR ‘Low Level Laser Therapy’:ab,ti OR ‘Low-Power Laser Irradiation’:ab,ti OR ‘Low Power Laser Irradiation’:ab,ti OR ‘Laser Biostimulation’:ab,ti OR ‘Biostimulation, Laser’:ab,ti OR ‘Laser Phototherapy’:ab,ti OR ‘Phototherapy, Laser’:ab,ti

8. Electric Stimulation Therapy

‘**Therapeutic Electrical Stimulation**’:ab,ti OR ‘**Electrical Stimulation, Therapeutic**’:ab,ti OR ‘**Stimulation, Therapeutic Electrical**’:ab,ti OR ‘**Therapeutic Electric Stimulation**’:ab,ti OR ‘**Electric Stimulation, Therapeutic**’:ab,ti OR ‘**Stimulation, Therapeutic Electric**’:ab,ti OR ‘**Electrical Stimulation Therapy**’:ab,ti OR ‘**Stimulation Therapy, Electrical**’:ab,ti OR ‘**Therapy, Electrical Stimulation**’:ab,ti OR ‘**Therapy, Electric Stimulation**’:ab,ti OR ‘**Stimulation Therapy, Electric**’:ab,ti OR ‘**Electrotherapy**’:ab,ti OR ‘**Interferential Current Electrotherapy**’:ab,ti OR ‘**Electrotherapy, Interferential Current**’:ab,ti

9. Extracorporeal Shockwave Therapy

‘Extracorporeal Shockwave Therapies’:ab,ti OR ‘Shockwave Therapies, Extracorporeal’:ab,ti OR ‘Shockwave Therapy, Extracorporeal’:ab,ti OR ‘Therapy, Extracorporeal Shockwave’:ab,ti OR ‘Shock Wave Therapy’:ab,ti OR ‘Shock Wave Therapies’:ab,ti OR ‘Therapy, Shock Wave’:ab,ti OR ‘Extracorporeal Shock Wave Therapy’:ab,ti OR ‘Extracorporeal High-Intensity Focused Ultrasound Therapy’:ab,ti OR ‘Extracorporeal High Intensity Focused Ultrasound Therapy’:ab,ti OR ‘HIFU Therapy’:ab,ti OR ‘HIFU Therapies’:ab,ti OR ‘Therapy, HIFU’:ab,ti OR ‘High-Intensity Focused Ultrasound Therapy’:ab,ti OR ‘High Intensity Focused Ultrasound Therapy’:ab,ti

10. **Amnion** /Biological Dressings

‘**placental membrane**’:ab,ti OR ‘**fetal membrane**’:ab,ti OR ‘**amniotic membrane**’:ab,ti OR ‘**amnion**’:ab,ti OR ‘**chorion**’:ab,ti OR ‘**Grafix**’:ab,ti OR ‘**EpiFix**’:ab,ti

**11. HBOT**

**'hyperbaric oxygenations':ab,ti OR 'oxygenations, hyperbaric':ab,ti OR 'hyperbaric oxygen therapy':ab,ti OR 'hyperbaric oxygen therapies':ab,ti OR 'oxygen therapies, hyperbaric':ab,ti OR 'oxygen therapy, hyperbaric':ab,ti OR 'therapies, hyperbaric oxygen':ab,ti OR 'therapy, hyperbaric oxygen':ab,ti OR 'oxygenation, hyperbaric':ab,ti**

**12.TOT**

**'topical oxygen':ab,ti OR 'topical wound oxygen':ab,ti OR 'continuous diffusion of oxygen':ab,ti OR 'continuous topical oxygen':ab,ti**

**13. ozone** (Excluded later due to eligibility criteria update)

**' ozone ':ab,ti OR ' ozon ':ab,ti**
