# Supplementary material for: Comparison of the efficacy of 12 interventions in the treatment of diabetic foot ulcers: a network meta-analysis
Source: PeerJ. 2025 Aug 11;13:e19809. doi: 10.7717/peerj.19809 (PMC12352421; doi:10.7717/peerj.19809)
Supplement: Supplemental Information 3 [file peerj-13-19809-s003.docx]

**Supplementary Table 1**. **League table of the** **ulcer healing rate**.

| **SC** |  |  |  |  |  |  |  |  |  |  |  |  |
| --- | --- | --- | --- | --- | --- | --- | --- | --- | --- | --- | --- | --- |
| 1.12 (0.45,2.76) | **AMT** |  |  |  |  |  |  |  |  |  |  |  |
| 1.36 (0.36,5.07) | 1.21 (0.37,3.94) | **LLLT** |  |  |  |  |  |  |  |  |  |  |
| 1.78 (0.69,4.59) | 1.59 (0.76,3.33) | 1.31 (0.39,4.40) | **EGF** |  |  |  |  |  |  |  |  |  |
| 1.96 (0.74,5.17) | 1.75 (0.81,3.78) | 1.44 (0.42,4.91) | 1.10 (0.49,2.48) | **ESWT** |  |  |  |  |  |  |  |  |
| 2.00 (0.77,5.23) | 1.79 (0.85,3.79) | 1.48 (0.43,5.02) | 1.12 (0.50,2.51) | 1.02 (0.44,2.36) | **NPWT** |  |  |  |  |  |  |  |
| 2.17 (0.86,5.47) | 1.94 (0.96,3.95) | 1.60 (0.49,5.26) | 1.22 (0.57,2.61) | 1.11 (0.51,2.43) | 1.08 (0.50,2.37) | **PRP** |  |  |  |  |  |  |
| 2.38 (0.99,5.76) | 2.13 (1.11,4.10) | 1.76 (0.55,5.58) | 1.34 (0.66,2.72) | 1.22 (0.58,2.54) | 1.19 (0.57,2.47) | 1.10 (0.56,2.16) | **TOT** |  |  |  |  |  |
| 2.60 (0.85,7.93) | 2.32 (0.90,5.99) | 1.91 (0.50,7.30) | 1.46 (0.54,3.90) | 1.33 (0.49,3.60) | 1.30 (0.47,3.55) | 1.20 (0.46,3.11) | 1.09 (0.44,2.72) | **LFU** |  |  |  |  |
| 2.58 (1.03,6.45) | 2.31 (1.15,4.62) | 1.90 (0.58,6.25) | 1.45 (0.68,3.07) | 1.31 (0.67,2.56) | 1.29 (0.60,2.75) | 1.19 (0.57,2.46) | 1.08 (0.55,2.13) | 0.99 (0.38,2.61) | **HBOT** |  |  |  |
| 3.03 (1.03,8.92) | 2.72 (1.10,6.68) | 2.24 (0.63,7.89) | 1.70 (0.66,4.37) | 1.55 (0.59,4.07) | 1.51 (0.58,3.94) | 1.40 (0.56,3.51) | 1.27 (0.53,3.07) | 1.17 (0.38,3.56) | 1.18 (0.47,2.94) | **ES** |  |  |
| 3.02 (1.20,7.58) | 2.70 (1.34,5.45) | 2.23 (0.68,7.30) | 1.70 (0.80,3.61) | 1.54 (0.71,3.35) | 1.51 (0.70,3.27) | 1.39 (0.67,2.87) | 1.27 (0.65,2.48) | 1.16 (0.45,3.03) | 1.17 (0.58,2.36) | 1.00 (0.40,2.49) | **PDGF** |  |
| 5.71 (2.64,12.34) | 5.11 (3.12,8.37) | 4.21 (1.44,12.29) | 3.20 (1.82,5.63) | 2.91 (1.61,5.28) | 2.85 (1.57,5.18) | 2.63 (1.57,4.41) | 2.40 (1.55,3.72) | 2.20 (0.99,4.91) | 2.22 (1.31,3.76) | 1.88 (0.87,4.05) | 1.89 (1.13,3.16) | **SOC** |

**Supplementary Table 2**. Node-splitting approach for inconsistency assessment of all comparisons.

| Side | Direct |  | Indirect |  | Difference |  | *P* |
| --- | --- | --- | --- | --- | --- | --- | --- |
|  | Coef | Std | Coef | Std | Coef | Std |  |
| A vs C | 1.50 | 0.56 | 0.39 | 2.24 | 1.10 | 2.30 | 0.631 |
| A vs D | 0.61 | 0.27 | 1.78 | 1.56 | -1.16 | 1.58 | 0.458 |
| A vs F | 0.74 | 0.36 | 1.90 | 0.58 | -1.16 | 0.69 | 0.092 |
| A vs H | 0.58 | 0.40 | 3.12 | 2.45 | -2.54 | 2.47 | 0.305 |
| C vs H | 0.13 | 1.22 | -1.16 | 0.76 | 1.29 | 1.44 | 0.369 |
| D vs K | -0.92 | 0.87 | 0.39 | 0.40 | -1.31 | 0.96 | 0.170 |
| F vs K | -0.89 | 0.50 | 0.27 | 0.48 | -1.16 | 0.69 | 0.092 |

Coef: coefficient; Std: standard deviation; IV: intravenous
